# Chinese Patent Medicine Shufeng Jiedu Capsules as an Adjuvant Therapy for Community-Acquired Pneumonia: A Systematic Review and Meta-Analysis of Randomized Clinical Trials

**DOI:** 10.3389/fphar.2022.923395

**Published:** 2022-07-04

**Authors:** Xiao-Wen Zhang, Ru-Yu Xia, Jia-Qi Gao, Jin-Jun Liu, De-Hao Xu, Xun Li, Xiao-Yang Hu, Merlin Willcox, Michael Moore, Meng-Yuan Dai, Jeanne Trill, Yu-Tong Fei, Jian-Ping Liu

**Affiliations:** ^1^ Centre for Evidence Based Chinese Medicine, Beijing University of Chinese Medicine, Beijing, China; ^2^ School of Qi-Huang Chinese Medicine, Beijing University of Chinese Medicine, Beijing, China; ^3^ Dongzhimen Hospital, Beijing University of Chinese Medicine, Beijing, China; ^4^ Primary Care, Population Sciences and Medical Education, University of Southampton, Southampton, England, United Kingdom; ^5^ Department of Respiratory and Critical Care Medicine, First Affiliated Hospital of Anhui Medical University, Hefei, China; ^6^ The National Research Center in Complementary and Alternative Medicine (NAFKAM) Department of Community Medicine, Faculty of Health Science, UiT, the Arctic University of Norway, Tromsø, Norway

**Keywords:** Chinese patent medicine, shufeng jiedu capsules, community-acquired pneumonia, systematic review, meta-analysis, randomized controlled trial

## Abstract

**Background:** Shufeng Jiedu (SFJD) capsules can be used as adjunctive treatment for patients with community-acquired pneumonia, but the effectiveness and safety of SFJD are not clear. This review aims to evaluate the effectiveness and safety of SFJD based on randomized controlled trials (RCTs).

**Methods:** A systematic review was conducted by searching PubMed, Embase, Scopus, Web of Science, CENTRAL, CNKI, VIP, CBM, Wanfang and trial registry platforms from their inception to March 2022. Two reviewers screened studies, extracted the data and assessed risk of bias independently. The data were pooled for meta-analysis or presented narratively.

**Results:** Seventeen RCTs involving 1840 participants were included. All trials compared SFJD plus antibiotics to antibiotics, or combined with symptomatic treatment in both groups. The overall certainty of evidence was assessed as moderate to very low certainty. Compared with routine treatment (antibiotics alone or antibiotics plus symptomatic treatment), SFJD plus routine treatment showed beneficial effects in resolution of fever (MD −1.20 days, 95%CI −1.73 to −0.67; 10 RCTs; very low certainty), cough (MD −1.02 days, 95%CI −1.23 to −0.81; 9 RCTs; moderate certainty), phlegm (MD −1.46 days, 95%CI −2.84 to −0.08; 6 RCTs; very low certainty), pulmonary crepitations (MD −1.61 days, 95%CI −2.64 to −0.59; 8 RCTs; low certainty), shortness of breath (MD −2.80 days, 95%CI −2.88 to −2.72; 2 RCTs; low certainty) and chest pain (MD −2.85 days, 95%CI −3.01 to −2.69; 1 RCT; low certainty). There was no significant difference in pathogen clearance (1 RCT). No serious adverse events were reported, but 2.60% (5/192) patients reported nausea in the SFJD groups, 1.04% (2/192) participants in routine group, and no significant difference was identified.

**Conclusions:** Current evidence suggests that adding SFJD may shorten the duration of symptom relief in community-acquired pneumonia for 1–2 days. The adverse events were minor and controllable, and no serious adverse events were reported. Well-reported trials and potential of reducing antibiotics were expected in the future studies.

## 1 Introduction

Community-acquired pneumonia (CAP) is an acute lung infection, that is, acquired outside of hospitals or other health care facilities, which may lead to significant morbidity, mortality, and cost (GBD 2017 Disease and Injury Incidence and Prevalence Collaborators, 2018). It is also one of the most common infectious diseases, accounting for 5–12% of lower respiratory infections (Brown, 2012; GBD 2015 LRI Collaborators, 2017). In the US, the age-adjusted incidence of CAP requiring hospital admission was 649 per 100,000 adults per year, corresponding to around 1.6 million hospitalizations (Ramirez et al., 2017). In China, the incidence of CAP was 713 per 100,000 per year (in all ages) (Sun et al., 2020). CAP can be caused by bacteria, viruses, fungi, or atypical bacteria. Bacteria are the most common cause of CAP, common organisms include *Streptococcus pneumoniae*, *Haemophilus influenzae*, *Klebsiella pneumoniae*, and *Staphylococcus aureus* (Shoar and Musher, 2020; Gan et al., 2022). Meanwhile, pandemics have focused attention on viral causes, such as severe acute respiratory syndrome (SARS), middle east respiratory syndrome (MERS), and coronavirus (COVID-19) (Nussbaumer-Streit et al., 2020; Yang et al., 2020). Children under 5 years and elderly adults over 65 years old are the most susceptible populations (World Health Organization, 2021).

The common initial treatment for CAP is anti-infective therapy, including empiric therapy, symptomatic treatment, and targeted therapy based on the pathogens. The choice of antibiotic would be tailored by the patient’s age, comorbidities, allergies, likely causative organism and antibiotic resistance patterns (Postma et al., 2015). Most guidelines recommend β-lactam antibiotics or macrolides for non-severe CAP, and β-lactam-macrolide or respiratory fluoroquinolones for severe CAP (World Health Organization, 2014; Lee et al., 2018; Metlay et al., 2019; NICE guideline, 2019; Barberán et al., 2021). However, the use of antibiotics may lead to adverse events and potential risks (NICE guideline, 2019; Huttner et al., 2020).

Shufeng Jiedu (SFJD) capsule, an oral Chinese patent medicine, was licensed as an over-the-counter drug by the National Medical Products Administration (NMPA) in July 2021 (NMPA, 2021), contains eight medicinal herbs. These have a range of reported therapeutic actions related to respiratory tract infections: *Bupleurum chinense DC.* [*Apiaceae*] (anti-infective, antipyretic), *Forsythia suspensa* (*Thunb.*) *Vahl* [*Oleaceae*] (anti-viral, cytotoxic), *Glycyrrhiza uralensis Fisch. ex DC.* [*Fabaceae*] (anti-infective, anti-inflammatory), *Isatis tinctoria subsp. tinctoria* [*Brassicaceae*] (anti-infective, eliminates toxins), *Patrinia scabiosifolia f. scabiosifolia* [*Caprifoliaceae*] (eliminates toxins), *Phragmites australis subsp. australis [Poaceae]* (immunomodulatory), *Reynoutria japonica Houtt.* [*Polygonaceae*] (antiviral)*, Verbena officinalis L.* [*Verbenaceae*] (anti-pyritic) (Simayi et al., 2022).

Pre-clinical research has corroborated that SFJD may have antibacterial, antiviral, anti-inflammatory, anti-pyretic and immunomodulatory effects (Bao et al., 2016; Xia et al., 2021; Ji et al., 2020; Li et al., 2017; Yuan et al., 2018; Yang et al., 2021; Tao et al., 2017). In a mouse model, SFJD acted as a broad-spectrum antimicrobial against gram positive and negative bacterial organisms, including *Staphylococcus aureus*, *Streptococcus pneumonia* and *Pseudomonas aeruginosa* (Bao et al., 2016). It was considered to reduce mortality due to *Staphylococcus aureus* by 26% and to *Streptococcus sp*. by 71%, compared with amoxicillin at 89 and 100% (Bao et al., 2016). SFJD was also found to reduce virus load, decrease inflammatory factors IL-6, IL-10, TNF-α, and IFN-γ in the lung of a coronavirus mouse model (Xia et al., 2021). Nevertheless, the SFJD doses (0.55, 1.10, and 2.20 g/kg) were higher than the control amoxicillin (2.75 ml/kg), suggesting that it may be a combination of pharmacological actions responsible for SFJD’s therapeutic activities.

In China, traditional Chinese medicine (TCM) is widely accepted by patients. Clinical evidence suggested that complementary medicine can be used to reduce antibiotic use in infection prevention and treatment (Baars et al., 2019). SFJD has been reported to have a positive effect on acute upper respiratory infections and acute exacerbation of chronic obstructive pulmonary disease (Xia et al., 2020; Zhang et al., 2021). Also, SFJD has been recommended by several TCM guidelines for CAP treatment (Xiong et al., 2016; Yu et al., 2019). However, there is a lack of systematic reviews of clinical evidence on SFJD for patients with CAP. Thus, this systematic review aims to evaluate the effectiveness and safety of SFJD as adjuvant therapy in CAP.

## 2 Materials and Methods

This review was reported according to the Preferred Reporting Items for Systematic Reviews and Meta-Analyses (PRISMA) (Page et al., 2021) and the registered protocol (Inplasy protocol 202060102. doi:10.37766/inplasy 2020.6.0102). The only deviation form the protocol is the searching time, we conducted a new search on 20 Mar 2022 before submitting, to make sure the evidence are latest and comprehensive.

### 2.1 Eligibility Criteria

Randomized controlled trials (RCTs) with participants at any age diagnosed as CAP (Chinese Thoracic Society, 2006; Metlay et al., 2019; Yu et al., 2019) were included. There were no restrictions on the gender, country or race of the participants. Quasi-RCTs and non-experimental studies were excluded due to their potential high risk of bias. We included trials which compared SFJD plus routine treatment (such as antibiotics, corticosteroids, physiotherapy or other regular treatment) with no treatment, placebo, routine treatment, or routine treatment plus placebo. We included trials which reported at least one expected outcome, incorporating the primary outcomes—resolution time of clinical symptoms, such as fever, cough, phlegm, focal inspiratory crepitations, etc.; and secondary outcomes—all-cause mortality, proportion of patients who had improvement on their chest radiograph, length of stay in hospital, duration and dosage of antibiotics use, treatment compliance, pathogen positive/negative rate, infection-related indices such as C-reactive protein (CRP) or procalcitonin (PCT), incidence of complications due to CAP, quality of life, and adverse events. The language of publication was not limited.

### 2.2 Date Sources and Search Terms

A search was carried out across the following databases: PubMed, Embase, Scopus, Web of Science, Cochrane Central Register of Controlled Trials (CENTRAL), China National Knowledge Infrastructure (CNKI), Chinese Scientific Journal Database (VIP), SinoMed, and Wanfang database from their inception to 20 Mar 2022. We also searched references of included studies, grey literature, and clinical trial registers, including ClinicalTrials.gov (https://clinicaltrials.gov), International Clinical Trials Registry Platform (www.who.int/ictrp/), and Chinese Clinical Trial Registry (https://www.chictr.org.cn/index.aspx). Also, the relevant experts provide suggestions to support the comprehensive search. Search strategies with different databases are in Supplementary Appendix A1.

### 2.3 Data Selection and Extraction

Two reviewers (JQG & JJL) independently screened the titles and abstracts of all potential studies. After preliminary screening, we retrieved the full-text of studies and two authors independently screened them. Disagreements were resolved through discussion with a third author (XWZ).

After completing the screening process, two reviewers independently extracted data including characteristics of the study, participants and diseases, details of interventions, outcome measures, and adverse events from all eligible trials.

### 2.4 Risk of Bias and Certainty of Evidence

Two reviewers (JQG & JJL) independently assessed the risk of bias for each study using the Cochrane Risk of Bias 2 tool (Sterne et al., 2019). Any disagreements were also resolved by discussion with the third reviewer (XWZ). Funnel plot tests for asymmetry were conducted to investigate potential publication bias if there were more than 10 trials in a single meta-analysis. The GRADE system was used to assess the certainty of the evidence for primary outcomes (Guyatt et al., 2008).

### 2.5 Data Synthesis

We pooled data with same comparison. Considering all the trials applied antibiotics, although several trials complemented with symptomatic treatment, we compared SFJD plus routine treatment with routine treatment. We estimated effect size using risk ratio (RR) with 95% confidence intervals (CI) for dichotomous data, mean difference (MD) with 95% CI for continuous data. Between-study heterogeneity was assessed using the I^2^ statistic. I^2^ > 30% represents moderate heterogeneity, I^2^ > 50% represents substantial heterogeneity and I^2^ > 75% represents considerable heterogeneity (Higgins et al., 2022). A fixed-effects model (FEM) was considered when I^2^ < 50%. Otherwise, a random-effects model (REM) was used.

To explain heterogeneity, we predefined subgroup analysis in terms of the severity of CAP (outpatient care, inpatient admission, or intensive care unit (ICU) admission), patient age (≤14 years old, 14–65 years old, ≥65 years old), and type of pathogen (bacterial, viral, fungal or atypical CAP).

Sensitivity analysis was performed to test the robustness of the results when there were clinically meaningful differences in primary outcomes by considering multi-center versus single center and risk of bias (by omitting studies that were judged to be at high risk of bias).

## 3 Results

### 3.1 Screening

**FIGURE 1:**
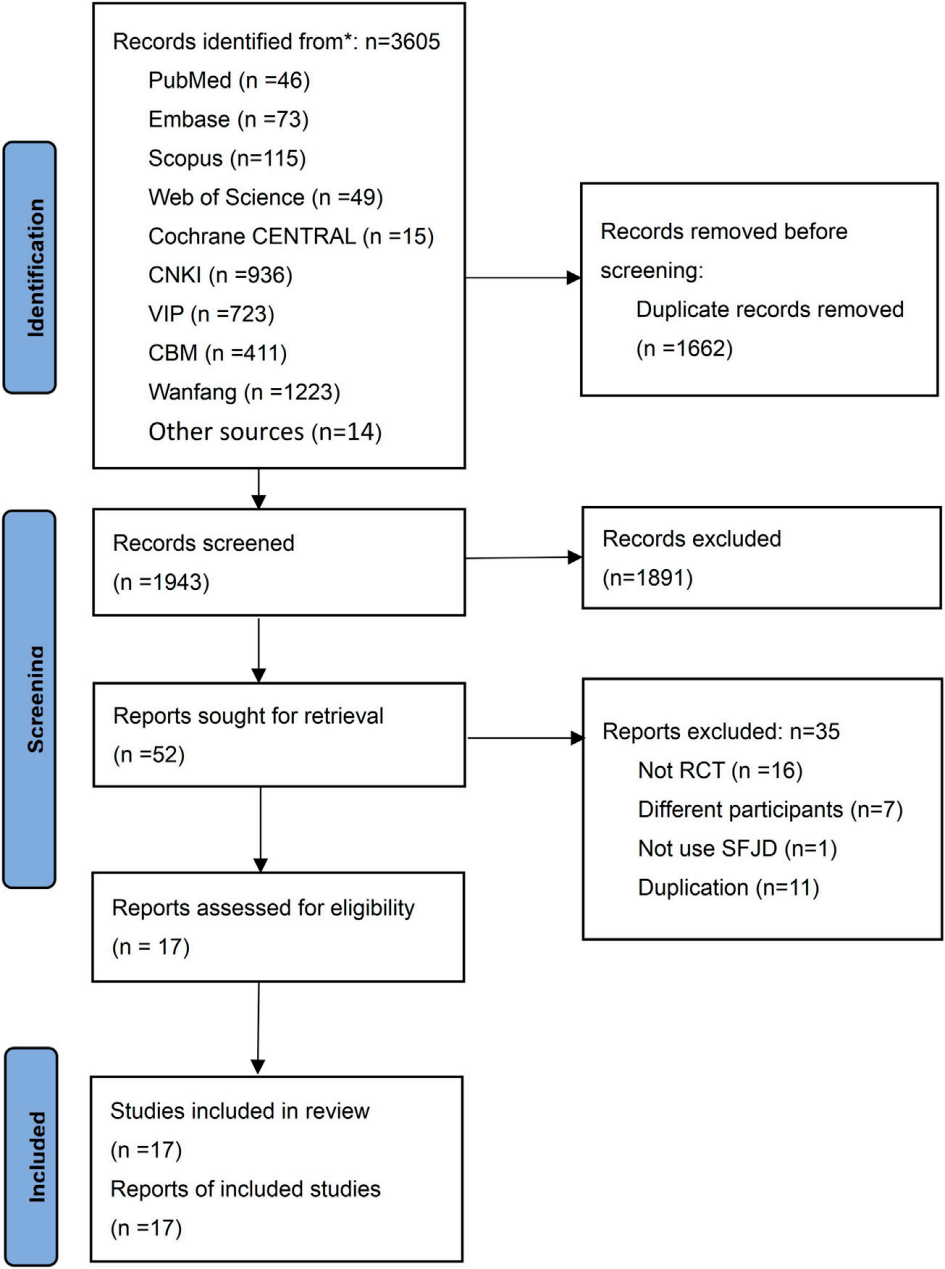
PRISMA flow chart of literature searching and screening.

We identified 3,605 potential studies initially, and 1,662 duplicates were removed. After reading the titles and abstracts, 1943 studies were excluded, and 52 studies were screened in full text. Finally, 17 RCTs (Zhang et al., 2014; Li et al., 2015; Zou, 2015; Wang, 2016; Wei et al., 2016; Yao and Liu, 2016; Zhang et al., 2016; Zhu and Li, 2016; Qu et al., 2019; Wu et al., 2019; Zhou et al., 2019; Guo et al., 2020; Li D. W. et al., 2020; Li X. Y. et al., 2020; Pan et al., 2020; Wang, 2020; Tang et al., 2021) involving 1840 participants were included in this study (Figure 1). The list of 35 excluded studies see Supplementary Appendix A2.

### 3.2 Characteristics of Included Studies

All 17 RCTs included were conducted in China and published in Chinese. One RCT focused on children from 1 to 11 years old (Zou, 2015), 1 RCT recruited all-age participants from children to elder (Wang, 2016), and the other 15 RCTs focused on adults, elders, or adults and elders. None of the trials reported the pathogens of participants. Except for one trial (Li D. W. et al., 2020) without reporting, the treatment duration of SFJD in all included trials ranged from 7 to 14 days, usually four capsules a time, three times per day, and reduction for children. Three trials only enrolled outpatients, and the other trials only recruited inpatients. The sample size ranged from 60 to 172 participants per trial. These participants had been diagnosed as CAP for 0–15 days before recruitment. Only two trials (Qu et al., 2019; Wang, 2020) reported funding information, which were supported by government funds. One trial (Yao and Liu, 2016) reported that 5 participants transferred to other hospitals during conducting so withdrew from the trial (Table 1). The summary of composition characteristics of preparations in all included articles see Supplementary Appendix A3.

**TABLE 1:** Characteristics of included randomized controlled trials.

Study ID	Sample size	Setting of Participants	Gender (male/female)		Age (years old)		Duration of symptoms before treatment/days		Intervention based on the control group	Routine treatment in both groups	Course of treatment (days)	Outcomes
			T	C	T	C	T	C				
Wang, (2020)	84	Inpatients	17/25	20/22	59.96 ± 8.65	58.35 ± 8.65	5.49 ± 1.87	5.52 ± 0.49	SFJD	Moxifloxacin (oral)	7	1, 5a, 5b, 5c, 8b, 8c, 8d
Wei et al., (2016)	120	Inpatients	32/28	31/29	57.64 ± 7.35	56.84 ± 8.13	NR	NR	SFJD	Moxifloxacin (iv)	7	1, 5b, 5c, 6, 7
Zhang et al., (2014)	100	Inpatients	NR	NR	60–75	60–75	NR	NR	SFJD	Moxifloxacin (iv)	7	1, 5a, 5b, 5c, 5d, 7
Pan et al., (2020)	120	Outpatients	36/24	32/28	39.81 ± 10.95	38.87 ± 10.28	2–4	2–5	SFJD	Moxifloxacin (iv)	10	2, 5e, 5f, 7
Wang, (2016)	128	Inpatients	40/24	38/26	30 ± 1.5	32 ± 2.5	NR	NR	SFJD	Cefuroxime Sodium (iv)	7	1, 5a, 5c, 5d, 5e, 7
Li et al., (2015)	60	Inpatients	12/18	14/16	53.1 ± 10.4	51.9 ± 11.3	3.7 ± 2.2	3.9 ± 1.9	SFJD	Cefuroxime Sodium (iv)	7	1, 2, 5a, 6, 7, 8a, 9
Wu et al., (2019)	172	Inpatients	0/86	0/86	38.63 ± 7.09	36.18 ± 8.10	4.94 ± 1.47	4.65 ± 1.83	SFJD	Sulbactam and Cefoperazone (iv)	7	1, 5a, 5b, 6, 7
Yao and Liu, (2016)	120	Inpatients	34/26	35/25	57.4 ± 8.5	58.6 ± 9.3	<2	<2	SFJD	Levofloxacin (iv)	7	1, 5a, 5b, 6, 7
Zhang et al., (2016)	84	Inpatients	NR	NR	20–72	20–72	NR	NR	SFJD	Levofloxacin (iv)	7	1, 6, 7
Zhu and Li, (2016)	120	Inpatients	27/33	29/31	43.5 ± 3.5	42.6 ± 3.8	NR	NR	SFJD	Levofloxacin (iv)	7–14	1, 2, 3, 5a, 5b, 5c, 5d, 6
Zou, (2015)	120	Outpatients	33/27	24/36	3.7 ± 1.3	3.5 ± 1.2	2.7 ± 1.5	2.5 ± 1.8	SFJD	Amoxicillin and clavulanate potassium (oral)	7	1, 7
Qu et al., (2019)	120	Inpatients	24/36	28/32	59.43 ± 18.12	58.50 ± 18.15	NR	NR	SFJD	Piperacillin Sodium and Tazobactam Sodium (iv)	7	1, 2, 5a, 5b, 5c, 5d, 6
Li D. W. et al., (2020)	120	Inpatients	36/24	39/21	49.71 ± 5.29	55.14 ± 3.73	1–10	1–10	SFJD	Antibiotcs by guidelines + symptomatic treatment	7	1, 2, 5c, 5e, 7
Zhou et al., (2019)	80	Inpatients	24/16	22/18	50.30 ± 6.40	48.56 ± 5.40	3.17 ± 0.88	3.44 ± 1.28	SFJD	Moxifloxacin (iv)+symptomatic treatment	7	1, 5b, 6, 7
Tang et al., (2021)	120	Inpatients	37/23	34/26	52.19 ± 5.25	53.23 ± 5.11	3.20 ± 0.90	3.25 ± 0.97	SFJD	Moxifloxacin (iv)+symptomatic treatment	7	1, 5a, 5b, 7
Li X. Y. et al., (2020)	80	Outpatients	36/4	38/2	69.58 ± 15.36	70.36 ± 13.69	11.47 ± 2.15	10.22 ± 2.97	SFJD	Moxifloxacin (oral)+symptomatic treatment	10	1, 5a, 5b
Guo et al., (2020)	92	Inpatients	28/18	25/21	69.85 ± 4.71	70.42 ± 4.88	6.57 ± 1.62	6.95 ± 1.70	SFJD	Cefuroxime Sodium (iv)+symptomatic treatment	10	1, 4

1. resolution time of clinical symptoms.

2. chest radiograph improvement.

3. length of stay in hospital.

4. pathogen negative rate.

5a. WBC, white blood cell; 5b. CRP, C-reactive protein; 5c. PCT, procalcitonin; 5d. NE, neutrophilicgranulocyte; 5e. IL-6, Interleukin-6; 5f. IL-8, Interleukin-8.

6. adverse events.

7. effective rate=(number of cured and improved participants/number of all participants) *100%.

8a. CPIS, Clinical pulmonary infection score; 8b. FEV1, Forced Expiratory Volume in the first second; 8c. FVC, forced vital capcacity; 8d. PEF, peak expiratory flow.

9. patients satisfaction.

T, trial group; C, control group; NR, not report; SFJD, shufeng jiedu capsules; TCM, traditional Chinese medicine; iv, intravenous.

### 3.3 Risk of Bias Assessment

**FIGURE 2:**
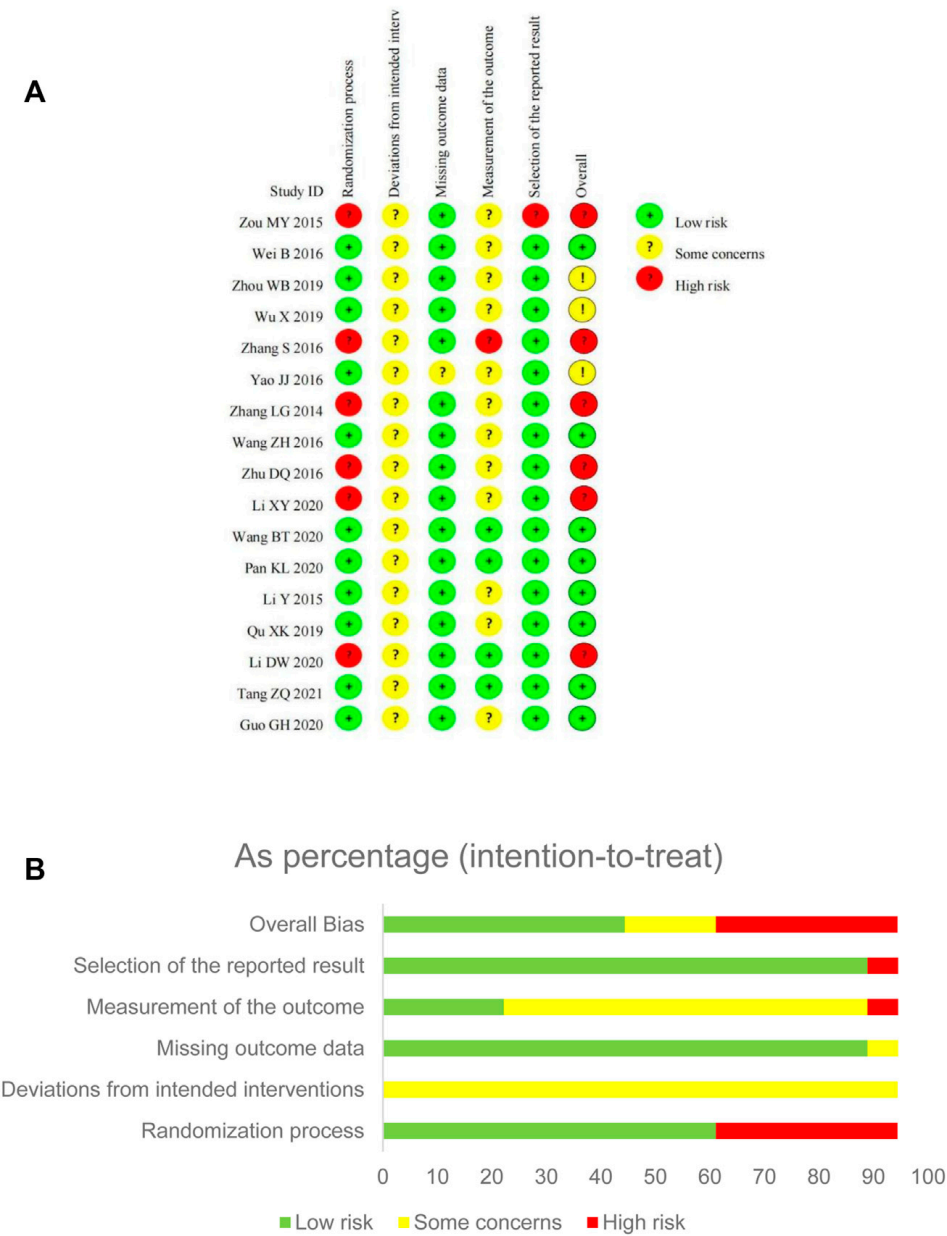
Risk of bias assessment for eligible studies. **(A)** Risk of bias summary; **(B)** Risk of bias graph.

For the overall bias, 9 RCTs were assessed as low risk, 3 RCTs as some concerns, and 6 RCTs as high risk. The risk of bias mainly arose from the randomization process, outcome measurement and reporting (Figure 2).

In the randomization process, more than half of the studies were judged as low risk bias as random number tables or software were utilized. All included studies showed no statistically significant difference between groups on baseline data. Some studies were judged as high risk of bias because of inadequate randomization, like the sequence of consulting the doctor (Zou, 2015), or just mentioned “random” without clear methods and concealment information (Zhang et al., 2014; Zhang et al., 2016; Zhu and Li, 2016; Li D. W. et al., 2020; Li X. Y. et al., 2020).

Although there was no placebo in any included trial, SFJD was commonly used as adjunctive treatment for CAP. All patients recruited signed the informed consent and agreed to accept the randomized treatment. Besides, the effect of participants in randomized groups were analyzed appropriately which would not affect the result. Due to the unclear reporting on blinding and appropriate analysis, all trials were judged as some concerns instead of high risk.

For the item of missing outcome data, only one trial (Yao and Liu, 2016) reported that 5 participants transferred to another hospital and withdrew from the study, which was assessed as some concerns. The other trials showed the same number of participants in the results as well as the baseline, so were assessed as low risk.

For the measurement of outcomes, the duration of symptoms focused on were usually self-reported by participants. This may have been influenced the by awareness of the intervention received, so 12 RCTs were judged as “some concerns”. In one trial (Zhang et al., 2016), we only collected its subjective outcomes-duration of symptoms and adverse events-for analysis, so it was judged as high risk.

For selection of the reported result, one trial (Zou, 2015) did not report protocol information, and the results reported were quite limited, so judged as high risk; the other 16 trials reported most of the expected outcomes comprehensively, so judged as low risk.

### 3.4 Primary Outcome

#### 3.4.1 Resolution Time of Fever

**FIGURE 3:**
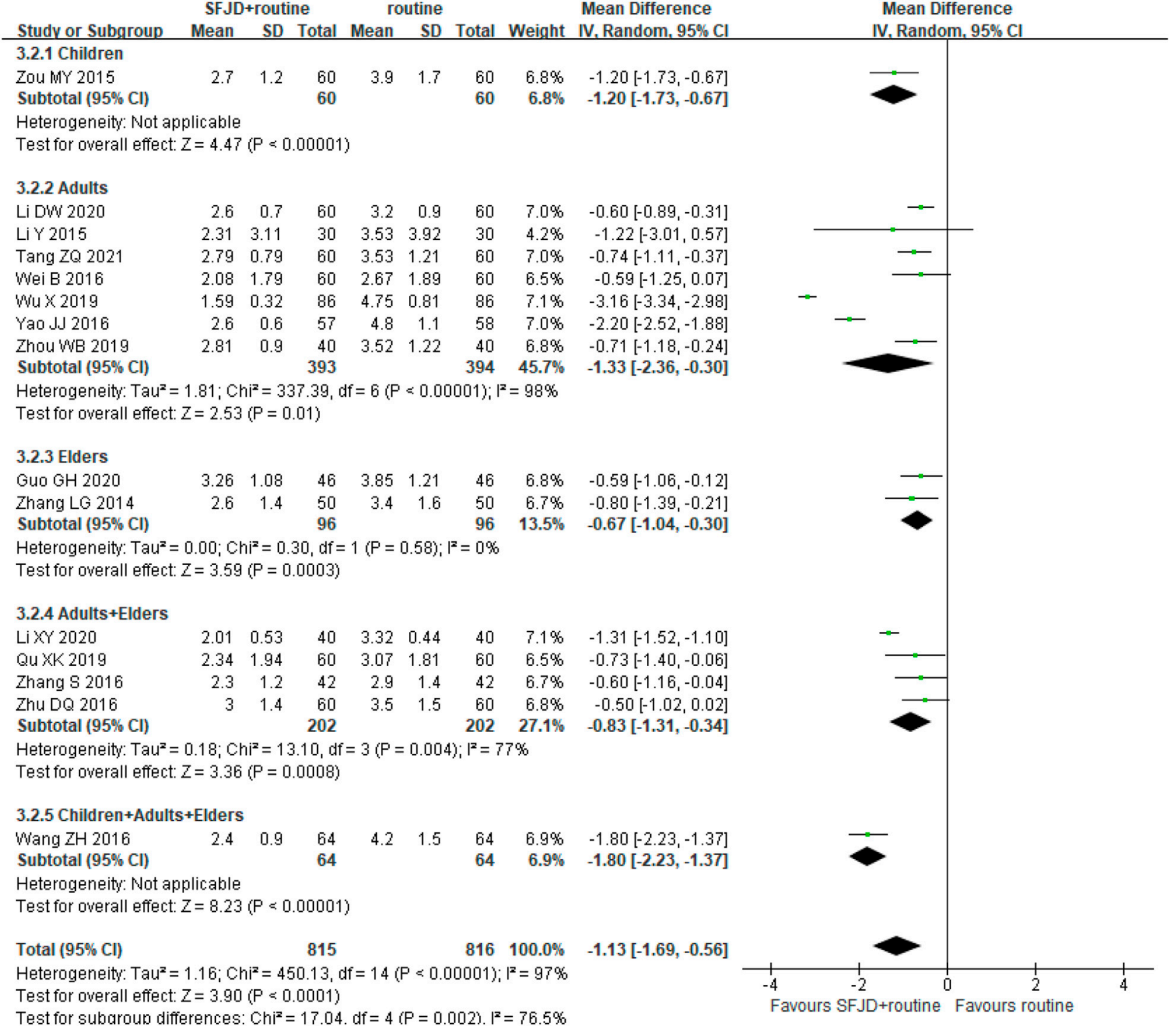
Forest plot of resolution time (days) of fever stratified by age. Comparison: SFJD plus routine treatment vs. routine treatment. SFJD: Shufeng Jiedu capsule.

By comparing SFJD plus routine treatment with routine treatment, 15 RCTs (1,631 participants) reported the duration of fever. The duration of fever was shorter in the SFJD group when compared with the control group (MD -1.13 days, 95%CI −1.69 to −0.56; I^2^ = 97%; REM; very low certainty) (Figure 3).

In subgroup analysis, the majority of these trials did not report the pathogen types, so we could only classify them by age or inpatient/outpatient status. Participants aged ≤ 14 years old were classified as children, 14–65 years old as adults, and ≥ 65 years old as the elders. Both showed high heterogeneity in the subgroups for this outcome, which may result from the different ways of drug administration, different age baseline among trails or other under-reported factors. Therefore, SFJD did not show specific effectiveness on populations at different ages.

#### 3.4.2 Duration of Cough

**FIGURE 4:**
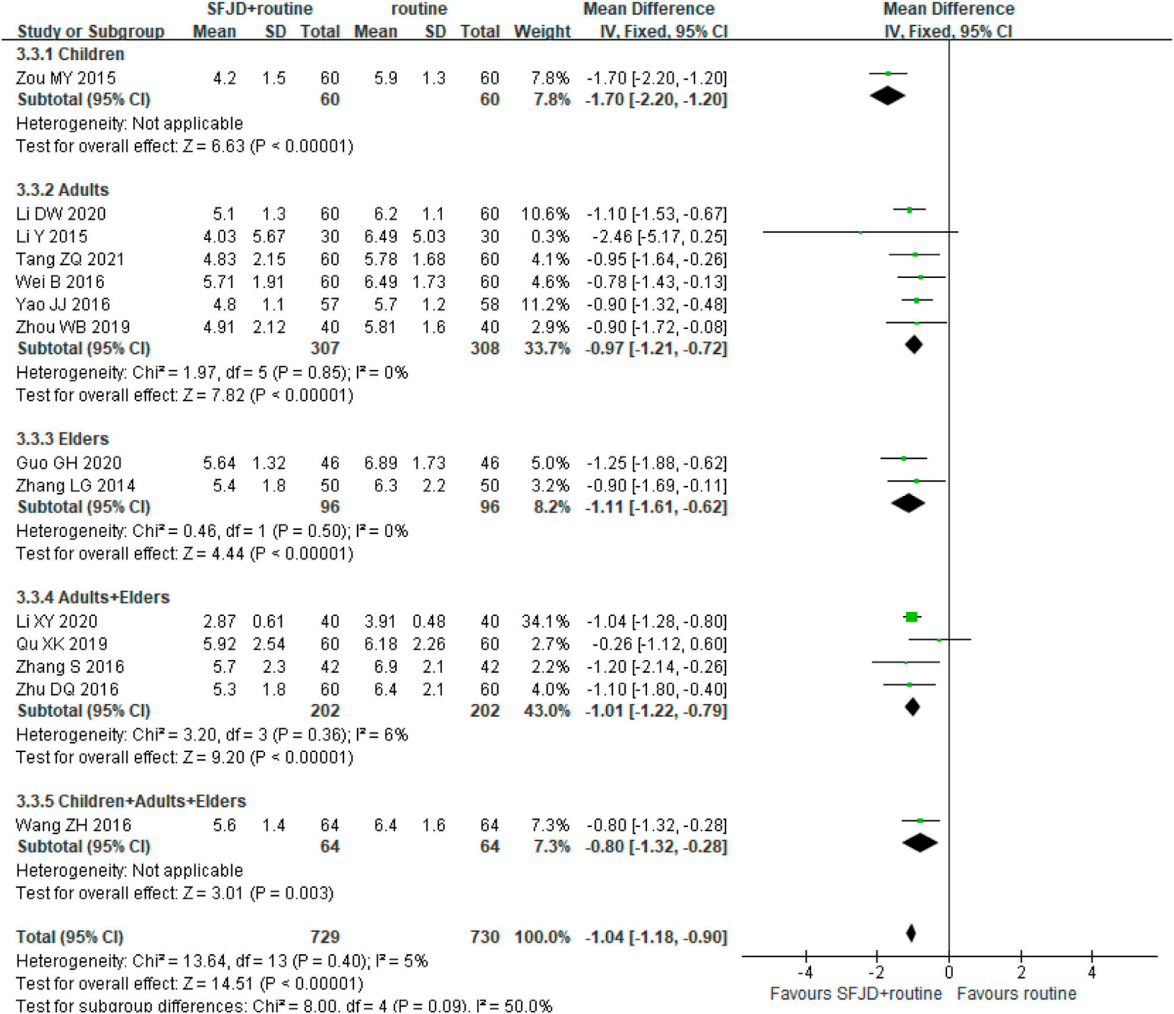
Forest plot of duration of cough (days) stratified by age. Comparison: SFJD plus routine treatment vs. routine treatment. SFJD: Shufeng Jiedu.

Fourteen trials (1,459 participants) reported the duration of cough. The duration in the SFJD group was significantly shorter than in the control group (MD −1.04 days, 95%CI −1.18 to −0.90; I^2^ = 5%; FEM; moderate certainty) (Figure 4). SFJD was effective in all age groups.

#### 3.4.3 Duration of Phlegm

Ten RCTs (1,124 participants) compared SFJD plus routine treatment with routine treatment alone on the duration of phlegm. had a significantly shorter duration of sputum production (MD −1.30 days, 95%CI −2.12 to −0.48; I^2^ = 98%; REM; very low certainty) (Supplementary Appendix A4).

#### 3.4.4 Duration of Pulmonary Crepitations

**FIGURE 5:**
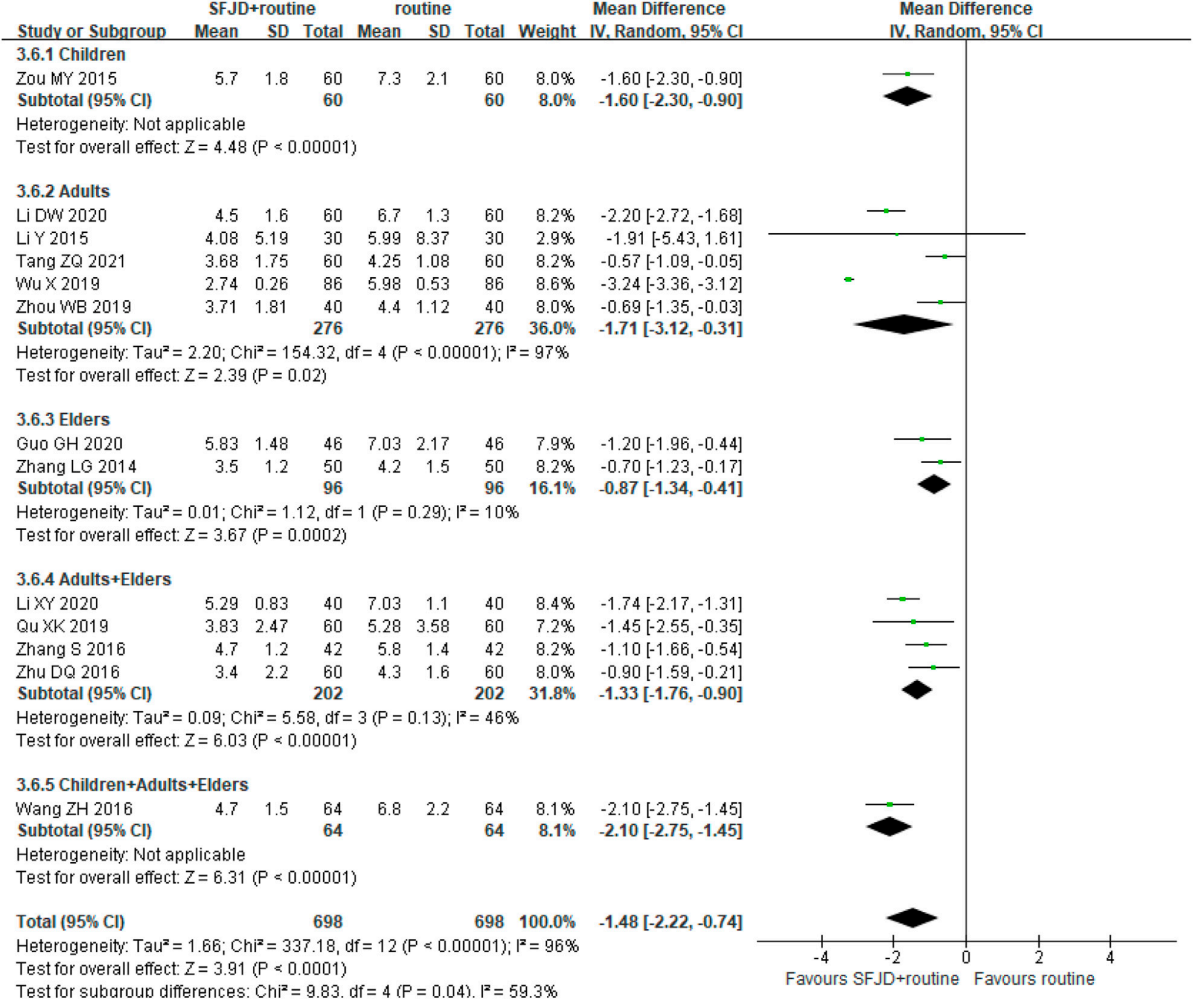
Forest plot of duration of pulmonary crepitations (days) stratified by age. Comparison: SFJD plus routine treatment vs. routine treatment. SFJD: Shufeng Jiedu.

Thirteen trials with 1,396 participants evaluated this outcome, and the SFJD group had a shorter duration of crepitations (MD −1.61 days, 95%CI −2.64 to −0.59; I^2^ = 96%; REM; low certainty). The age of participants varied, and high heterogeneity was observed in the subgroups, which may result from the small sample size. SFJD did not demonstrate specific effectiveness on populations at different ages (Figure 5).

#### 3.4.5 Duration of Shortness of Breath

Two trials with 232 participants compared SFJD plus antibiotics with antibiotics alone, all trials showed the SFJD group had a shorter duration of shortness of breath than the control group (MD −2.80 days, 95%CI −2.88 to −2.72; I^2^ = 0%; low certainty) (Supplementary Appendix A5).

#### 3.4.6 Duration of Chest Pain

Only one RCT (Wu et al., 2019) involving 172 adults reported this outcome. This trial compared SFJD plus antibiotics with antibiotics alone. Duration of chest pain in the SFJD group was 3.20 ± 0.43 days, and in control group was 6.05 ± 0.61 days (MD −2.85 days, 95%CI −3.01 to −2.69; *p* < 0.00001; low certainty) (Supplementary Appendix A6).

### 3.5 Secondary Outcomes

No included trial reported all-cause mortality, treatment compliance, incidence of complications due to CAP or quality of life.

#### 3.5.1 Improvement Rate of Chest Radiograph

Five trials (540 participants) reported the improvement of chest radiographs. Four trials applied CT imaging to observe the absorption of the inflammation, and one trial (Li et al., 2015) applied x-rays. After treatment for about 7 days, 156 more per 1,000 people in the SFJD group showed improvement in their radiograph (RR 1.21, 95%CI 1.12 to 1.31; I^2^ = 0%; FEM; moderate certainty) (Supplementary Appendix A7).

#### 3.5.2 Length of Hospital Stay

Only one of the included trials (Zhu and Li, 2016) reported this outcome. Patients were hospitalized for 6.1 ± 2.3 days when treated with SFJD plus levofloxacin, and hospitalized for 8.1 ± 2.3 days when treated with levofloxacin alone (*p* < 0.05).

#### 3.5.3 Duration and Dosage of Antibiotics Use

None of the included trials reported this as an outcome. All the trials reported the duration and dosage of antibiotics in methods, and kept the same dosage in the whole course of treatment.

#### 3.5.4 Pathogen Clearance

Only one trial (Guo et al., 2020) with 92 elderly participants reported this outcome. The trial compared SFJD plus Cefuroxime Sodium with Cefuroxime Sodium, combined with symptomatic treatment in both groups. Researchers cultured the sputum and isolated the pathogens. After 10 days treatment, the SFJD group cleared 86.96% (40/46 strains) pathogen, and control group cleared 81.82% (36/44 strains) pathogen. There were no significant differences between the two groups (*p* > 0.05).

#### 3.5.5 Infection Indices: White Cell Count

**FIGURE 6:**
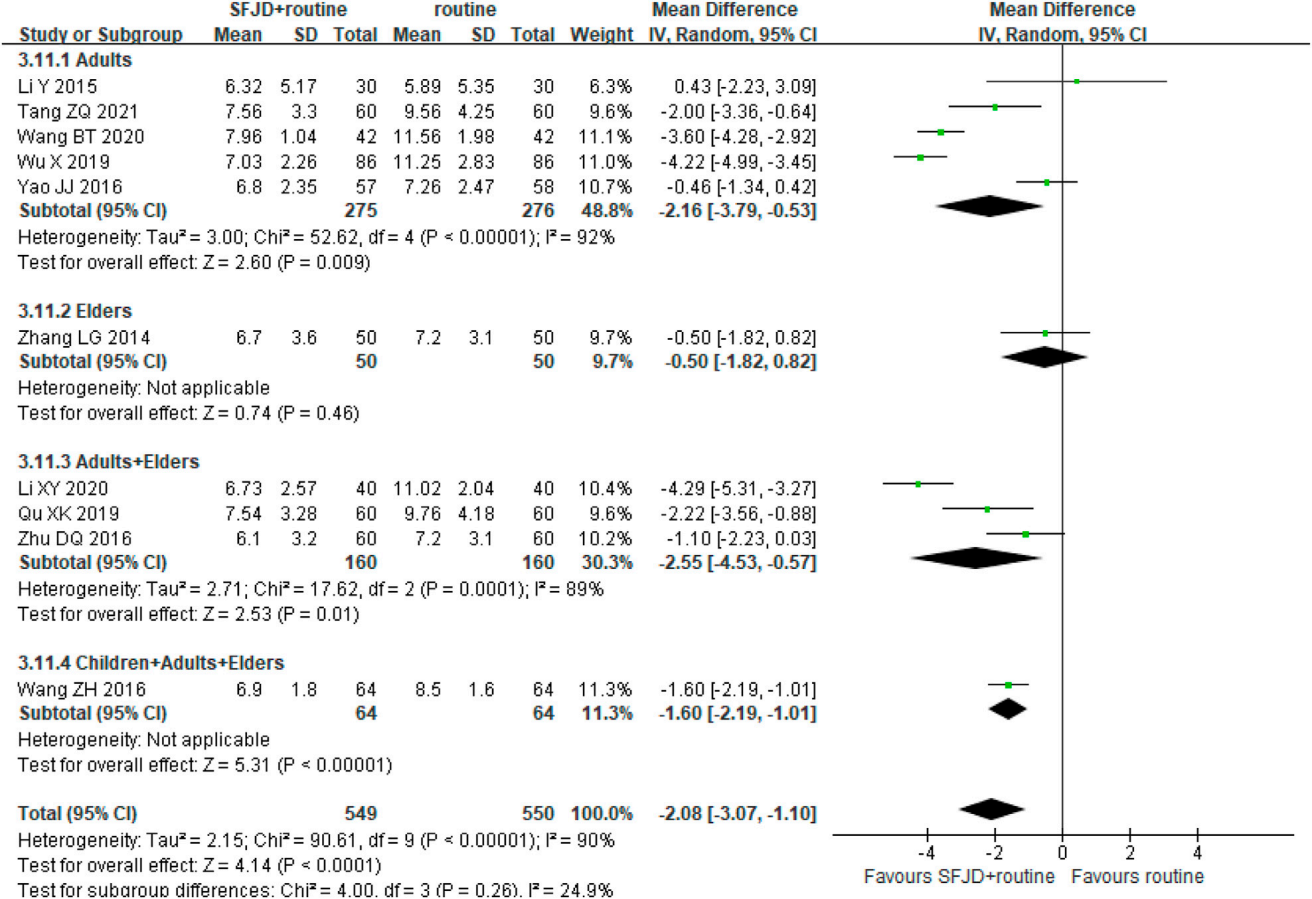
Forest plot of white cell count stratified by age (×10^9^/L). Comparison: SFJD plus routine treatment vs. routine treatment. SFJD: Shufeng Jiedu.

Ten trials (1,099 participants) counted WCC before and after the treatment. After the treatment, the WCC in the SFJD group reduced more than that in the control group (MD −2.08 × 10^9^/L, 95%CI −3.07 to −1.10; REM; I^2^ = 90%; very low certainty). With subgroup analysis, we found that SFJD may be effective for the adults on WCC, and no significant difference was found in the elders group (Figure 6).

#### 3.5.6 Infection Indices: C-Reactive Protein

**FIGURE 7:**
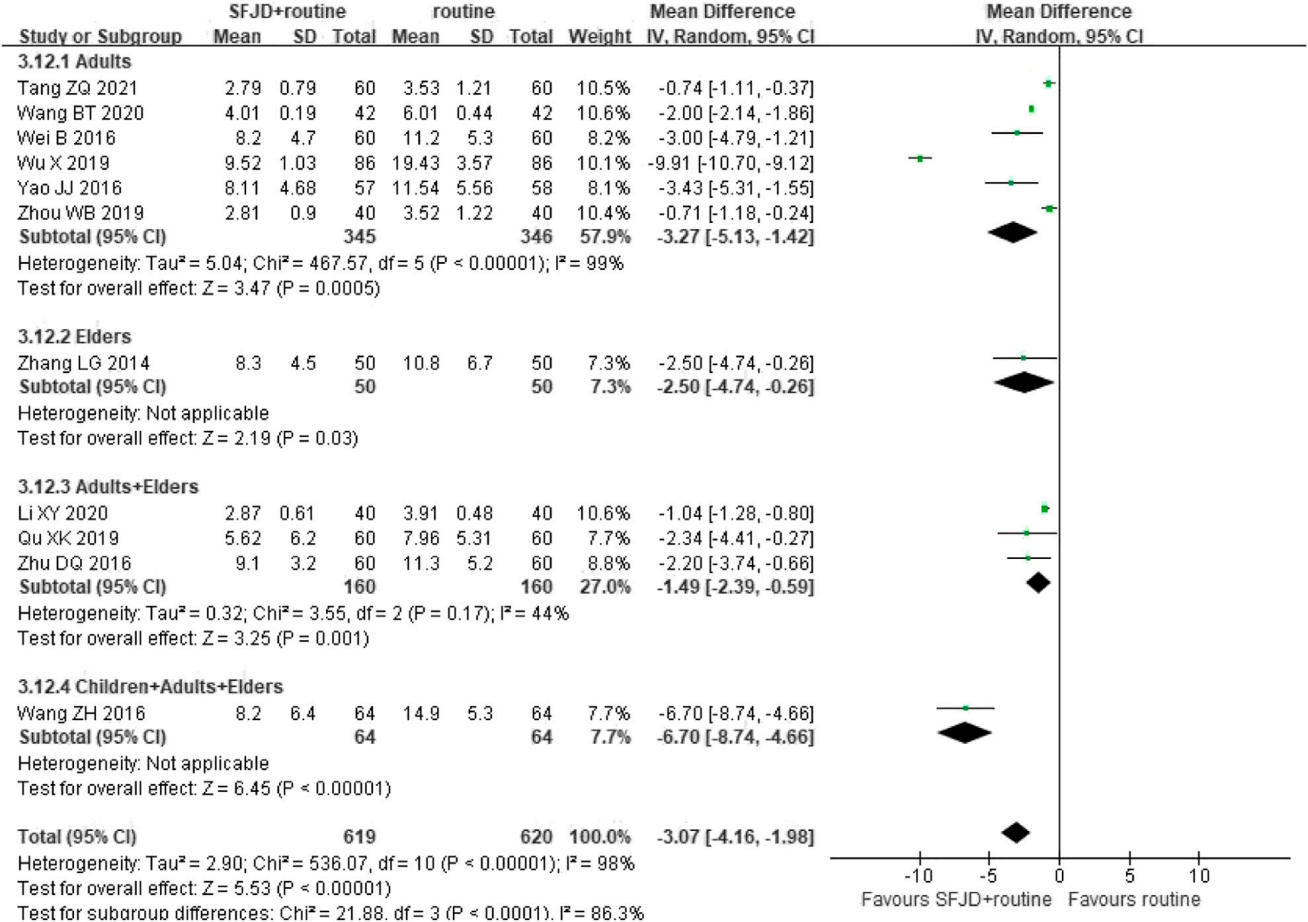
Forest plot of C-reactive protein stratified by age (mg/L). Comparison: SFJD plus routine treatment vs. routine treatment. SFJD: Shufeng Jiedu.

Eleven RCTs (1,239 participants) reported the a reduction in CRP. The pooled data indicated that CRP of patients in SFJD group reduced more than that in control group (MD −3.07 mg/L, 95%CI −4.16 to −1.98; I^2^ = 98%; REM; low certainty). With subgroup analysis, SFJD showed effectiveness in all age groups. (Figure 7).

#### 3.5.7 Infection Indices: Procalcitonin

Seven RCTs (792 participants) reported PCT levels. The trials varied in antibiotic and had high heterogeneity, which may due to the different starting value and time of drug onset, and there was no significant difference in the PCT level between the SFJD and antibiotics groups (MD −0.29 ng/ml, 95%CI −0.68 to 0.09; I^2^ = 100%; REM; very low certainty) after treatment (Supplementary Appendix A8).

#### 3.5.8 Adverse Events

Eight studies reported adverse events, among which four studies (Zhu and Li, 2016; Qu et al., 2019; Wu et al., 2019; Zhou et al., 2019) declared there was no adverse event in both groups during the treatment, and the other four studies (Li et al., 2015; Wei et al., 2016; Yao and Liu, 2016; Zhang et al., 2016) reported the cases of specific events (Table 2). For the most reported adverse event-nausea, 2.60% (5/192) patients reported in the SFJD groups, 1.04% (2/192) participants in routine group, and no significant difference was identified (RR 2.00, 95%CI 0.51 to 7.88; I^2^ = 0%; FEM; low certainty) (Supplementary Appendix A9).

**TABLE 2:** Adverse events reported in included RCTs on SFJD for community-acquired pneumonia.

Study ID	T	C
Wei et al., (2016)	2 nausea	1 abdominal pain
Li et al., (2015)	2 RBC in urine routine; 1 nausea	1 RBC in urine routine
Yao and Liu, (2016)	1 nausea	1 nausea
Zhang et al., (2016)	1 uncomfortable in abdomen and and nausea; 1 phlebitis	2 abdominal distension and nausea; 1 vomiting; 1 phlebitis

T, experimental group; C, control group; RBC, red blood cell.

### 3.6 Subgroup Analysis

Age of participants and severity of CAP (inpatients/outpatients) did not show interaction on the results.

### 3.7 Sensitivity Analysis

For the primary outcomes with positive results, no significant change was found after deleting trials with high risk of bias in sensitivity analysis (Supplementary Appendix A10).

### 3.8 Publication Bias

**FIGURE 8:**
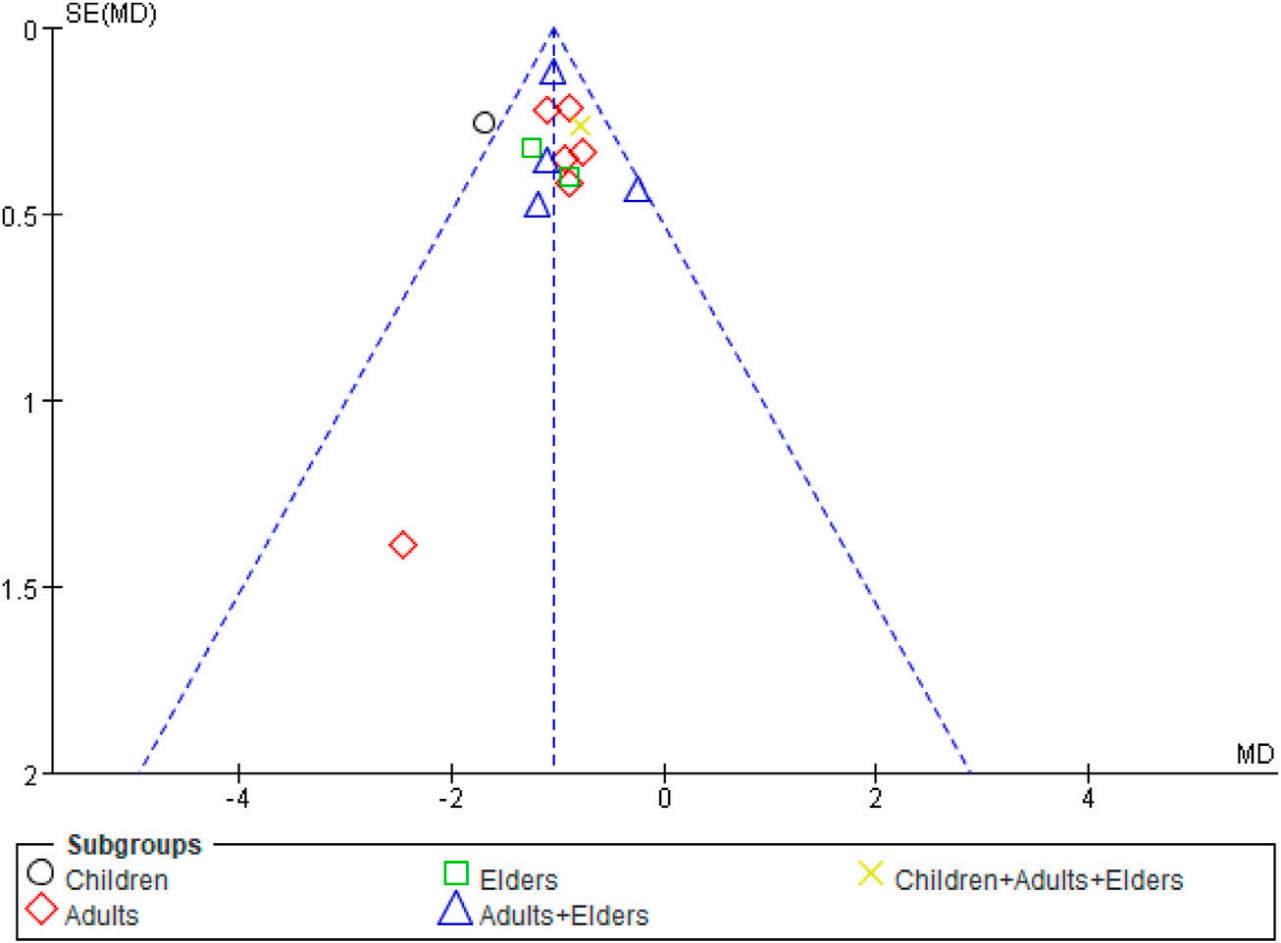
Funnel plot of comparison: SFJD plus routine treatment vs. routine treatment, outcome: duration of cough. SFJD: Shufeng Jiedu.

For each group of the comparative analysis with more than 10 trials, we conducted the funnel plot. Only one outcome, the duration of cough, showed a similar symmetric funnel plot (Figure 8). For the other outcomes including duration of fever, duration of pulmonary crepitations, and CRP, the funnel plots see Supplementary Appendix A11.

### 3.9 Certainty of Evidence

The GRADE tool was applied to assess the certainty of evidence for each outcome. We downgraded the certainty of evidence for each outcome considering its risk of bias, inconsistency, indirectness, imprecision and other potential bias. The majority of evidence was assessed as moderate to very low certainty (Table 3).

**TABLE 3:** Certainty of the evidence according to GRADE, question: Shufeng Jiedu capsules plus routine treatment compared to routine treatment for patients with community-acquired pneumonia.

Certainty assessment							No. of patients		Effect		Certainty
No. of studies	Study design	Risk of bias	Inconsistency	Indirectness	Imprecision	Other considerations	SFJD + routine	routine	Relative (95% CI)	Absolute (95% CI)	
Resolution time of fever											
15	randomized trials	serious	serious	not serious	serious	not serious	815	816	-	MD 1.13 days lower (1.69 lower to 0.56 lower)	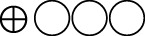 Very low
Duration of cough											
14	randomized trials	serious	not serious	not serious	not serious	not serious	729	730	-	MD 1.04 days lower (1.18 lower to 0.9 lower)	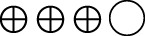 Moderate
Duration of phlegm											
10	randomized trials	serious	serious	not serious	serious	not serious	562	562	-	MD 1.3 days lower (2.12 lower to 0.48 lower)	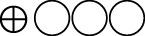 Very low
Duration of pulmonary crepitations											
13	randomized trials	serious	serious	not serious	not serious	not serious	698	698	-	MD 1.48 days lower (2.22 lower to 0.74 lower)	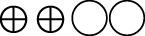 Low
Duration of shortness of breath											
2	randomized trials	serious	not serious	not serious	serious	not serious	116	116	-	MD 2.8 days lower (2.88 lower to 2.72 lower)<	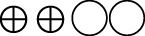 Low
Duration of chest pain											
1	randomized trials	serious	not serious	not serious	serious	not serious	86	86	-	MD 2.85 days lower (3.01 lower to 2.69 lower)	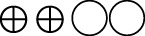 Low
Improvement rate of chest radiograph											
5	randomized trials	serious	not serious	not serious	not serious	not serious	247/270 (91.5%)	204/270 (75.6%)	RR 1.21 (1.12–1.31)	159 more per 1,000 (from 91 more to 234 more)	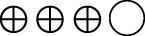 Moderate
White cell count (WCC)											
10	randomized trials	serious	serious	not serious	serious	not serious	549	550	-	MD 2.08 × 109/L lower (3.07 lower to 1.1 lower)	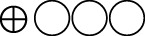 Very low
C-reactive protein (CRP)											
11	randomized trials	serious	serious	not serious	not serious	not serious	619	620	-	MD 3.07 mg/L lower (4.16 lower to 1.98 lower)	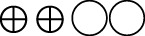 Low
Procalcitonin (PCT)											
7	randomized trials	serious	serious	not serious	serious	not serious	396	396	-	MD 0.29 ng/ml lower (0.68 lower to 0.09 higher)	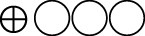 Very low
Adverse event-nausea											
4	randomized trials	serious	not serious	not serious	serious	not serious	5/192 (2.6%)	2/192 (1.0%)	RR 2.00 (0.51–7.88)	2 more per 1,000 (from 1 more to 8 more)	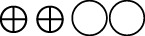 Low

CI, confidence interval; MD, mean difference; RR, risk ratio.

## 4 Discussion

### 4.1 General Interpretation of the Results

This review included 17 RCTs involving 1840 participants on SFJD for CAP. The trials were assessed as moderate to very low certainty by GRADE, and the risk of bias was mainly due to the randomization process, as well as deviation from intended interventions and measurement of the outcomes due to lack of blinding. When compared with routine treatment, SFJD may help reduce the patients symptoms, such as incorporating fever, cough, phlegm, pulmonary crepitations, shortness of breath and chest pain, by 1 day. SFJD may not improve pathogen clearance or PCT, and the evidence was limited. In addition, SFJD may be helpful in improving chest radiograph and resolving inflammation indices, like WCC, CRP. According to the limited evidence, SFJD showed no significant difference in any adverse event, and no serious adverse events were reported.

### 4.2 Comparison With Previous Studies

Systematic reviews of SFJD (Xia et al., 2020; Zhang et al., 2021) for other respiratory diseases support the improvement of clinical symptoms. For participants with acute exacerbation of chronic obstructive pulmonary disease, five trials showed that SFJD may reduce the time of sputum, crackles and cough. For acute upper respiratory tract infections, they found SFJD may shorten the duration of fever, cough and sore throat. These findings were consistent with our review, which demonstrated that SFJD seems to play a positive role in symptom relief, including fever, cough, phlegm, crepitations, shortness of breath and chest pain. Although no included studies reported the quality of life in patients, the improvement of severity and duration of symptoms probably benefit the life experience as well as mental health of patients.

Several studies also showed SFJD could improve infectious indices. An experiment in rats (Liao et al., 2021) showed that SFJD alleviates the inflammatory response in lung injury via the NRF2-associated antioxidant pathway. For the current COVID-19 pandemic, studies showed SFJD owned the antiviral and anti-inflammatory properties (Xia et al., 2021), and the active ingredients of SFJD regulates the immune system and anti-inflammatory related targets on multiple pathways (Tao et al., 2020). Moreover, SFJD was recommended in national treatment guidelines for COVID-19 as well as CAP in China (National Health Commission of the People’s Republic of China and National Administration of Traditional Chinese Medicine, 2020; Yu et al., 2019; Xiong et al., 2016).

For the safety of SFJD, studies showed no serious adverse events were reported (Zhang et al., 2021) and there were no significant differences between the SFJD and control groups (Xia et al., 2020). Nausea was reported in a few cases. An overview of systematic reviews (Xu et al., 2022) of SFJD found that the main side-effects of SFJD were gastrointestinal discomfort (including nausea, diarrhea and vomiting), but the incidence of these events was low, and could be relieved after stopping administration.

### 4.3 Strengths and Limitations

We have conducted a comprehensive research including English and Chinese databases. The majority of included trials reported the duration of symptoms, which were our expected primary outcomes. Besides, all eligible trials were evaluated with proper methods as well as assessment tool.

However, our review has limitations. First, these included trials did not fully report all our expected outcomes, rare report on pathogens of pneumonia, all-cause mortality, duration and dosage of antibiotics use, treatment compliance of participant, incidence of complications due to CAP, quality of life. Secondly, the description of randomization process and design of placebo or blinding were insufficient, and several trials were assessed as low or very low certainty, which led to our cautious attitude of the results. Third, although we did not limit the region of trial conduct nor the language of published articles, due to the approval limitation of SFJD, only Chinese trials were included, which may limit the generalizability of our findings.

### 4.4 Implications

For the design of the future trials, placebo controlled RCTs are recommended, which could realize the blinding of participants and researchers, and improve the quality of clinical trials. Besides, SFJD tend to be a potential intervention for supporting antibiotic stewardship by reducing the duration of antibiotic treatment, so the future trials were recommended to focus on this point. Future RCTs should report a wide range of objective variables during treatment, like the duration and dosage of antibiotic treatment, which could provide more details for the outcomes evaluation as well as the good practice statement or guidelines, and may benefit for reducing antibiotic resistance. In addition, randomized controlled trials should use reporting criteria, like CONSORT (Schulz et al., 2011), in order that the methods of randomization or concealment could be observed. All in all, high-quality evidence could support a high-certainty conclusion on effectiveness and safety of SFJD for CAP. These implications are also applicable for most clinical trials of TCM.

## 5 Conclusion

Compared with routine treatment, SFJD plus rountine treatment may reduce the duration of clinical symptoms by 1–2 day, for fever, cough, phlegm, crepitations. It may improve the resolution of chest radiograph changes and infectious indices, but there was no significant effect on pathogen clearance and PCT based on the included seventeen RCTs. The adverse events were minor and controllable, and no serious adverse events were reported. Future placebo controlled trials should be conducted and reported to a high level of quality, for example, reporting comprehensively and transparently on the randomization process, blinding the patients as well as researchers if practicable. Besides, it would be promising for the future clinical trials to see whether and how SFJD could support antibiotic stewardship by enabling a significantly reduced course of antibiotics.

## Data Availability

The original contributions presented in the study are included in the article/Supplementary Material, further inquiries can be directed to the corresponding author.
