# Supplementary material for: Chinese Patent Medicine Shufeng Jiedu Capsules as an Adjuvant Therapy for Community-Acquired Pneumonia: A Systematic Review and Meta-Analysis of Randomized Clinical Trials
Source: Front Pharmacol. 2022 Jul 4;13:923395. doi: 10.3389/fphar.2022.923395 (PMC9289368; doi:10.3389/fphar.2022.923395)
Supplement: Supplementary file 1 [file DataSheet1.pdf]

Supplementary Appendix A1：Search strategies

Databases	Search terms
English search strategy	#1 Shufengjiedu
	#2 Shu Feng Jie Du
	#3 Shu-Feng-Jie-Du
	#4 Shufeng Jiedu
	#5 Shufeng-Jiedu
	#6 SFJD
	#7 #1 OR #2 OR #3 OR #4 OR #5 OR #6
Chinese search strategy	#1 疏风解毒胶囊 (Shufeng Jiedu Jiaonang)
	#2 疏风解毒 (Shufeng Jiedu)
	#3 #1 OR #2

Supplementary Appendix A2：List of excluded studies

Excluded reasons	Citations
Not RCT<br>(n =16)	1. Chen, L., Cheng, Z. Q., Liu, F., Xia, Y., & Chen, Y. G. (2020). [Analysis of 131 cases of COVID-19 treated with Ganlu Xiaodu Decoction]. [Journal Article]. *Zhongguo Zhong Yao Za Zhi*, 45(10), 2232-2238. doi: 10.19540/j.cnki.cjcmm.20200322.505<br>2. Chen, J., Lin, S., Niu, C., & Xiao, Q. (2020). Clinical evaluation of Shufeng Jiedu Capsules combined with umifenovir (Arbidol) in the treatment of common-type COVID-19: a retrospective study. *Expert Review of Respiratory Medicine*. doi: 10.1080/17476348.2020.182274<br>3. Qu, X. K., Hao, S. L., Ma, J. H., Wei, G. Y., Song, K. Y., Tang, C.,... Du W., J. (2020). Observation on clinical effect of Shufeng Jiedu Capsule combined with Arbidol Hydrochloride Capsule in treatment of COVID-19. *Chinese Traditional and Herbal Drugs*, 51(5), 1167-1170. doi: 10.7501/j.issn.0253-2670.2020.05.011<br>4. Qu, X. K., Hao, S. L., Ma, J. H., Wei, G. Y., Song, K. Y., Tang, C.,... Du, W. J. (2020). Observation on clinical effect of Shufeng Jiedu Capsule combined with Arbidol Hydrochloride Capsule in treatment of COVID-19. *Chinese Traditional and Herbal Drugs*, 51(5), 1167-1170. doi: 10.7501/j.issn.0253-2670.2020.05.011<br>5. Wang, Z., Chen, X., Lu, Y., Chen, F., & Zhang, W. (2020). Clinical characteristics and therapeutic procedure for four cases with 2019 novel coronavirus pneumonia receiving combined Chinese and Western medicine treatment. *BIOSCIENCE TRENDS*, 14(1), 64-68. doi: 10.5582/bst.2020.01030<br>6. Chen, L., ... Chen, Y. G. (2020). Clinical Efficacy of Shufeng Jiedu Capsule Combined with Western Medicine in Treatment of Common COVID-19 Patients by Retrospective Analysis[回顾性分析疏风解毒胶囊联合西医治疗普通型新型冠状病毒肺炎患者的临床疗效]. *Chinese Journal of Experimental Traditional Medical Formulae[中国实验方剂学杂志]*.<br>7. Qu, X. K.,... Du W., J. (2020). Observation on clinical effect of Shufeng Jiedu Capsule combined with Arbidol Hydrochloride Capsule in treatment of COVID-19[疏风解毒胶囊联合阿比多尔治疗新型冠状病毒肺炎的回顾性研究]. *Chinese Traditional and Herbal Drugs*, 51(05), 1167-1170<br>8. Qu, X. K., Hao, S. L., Ma, J. H., Wei, G. Y., Song, K. Y., Tang, C.,... Du, W. J. (2020). Observation on clinical effect of Shufeng Jiedu Capsule combined with Arbidol Hydrochloride Capsule in treatment of COVID-19[疏风解毒胶囊联合阿比多尔治疗新型冠状病毒肺炎的回顾性

	研究]. *Chinese Traditional and Herbal Drugs.*<br>9. Xiao, Q., Jiang, Y. j., Wu, S. S., ... Wu, J. J. (2020). Value analysis of Chinese medicine Shufeng Jiedu capsules combined with Abidol for mild COVID-19[中药疏风解毒胶囊联合阿比多尔治疗轻症新型冠状病毒肺炎的价值分析]. *Journal of Emergency in Traditional Chinese Medicine[中国中医急症]*, 29(05), 756-758<br>10. Xiao, Q., Jiang, Y. j., Wu, S. S., ... Wu, J. J. (2020). Value analysis of Chinese medicine Shufeng Jiedu capsules combined with Abidol for mild COVID-19[中药疏风解毒胶囊联合阿比多尔治疗轻症新型冠状病毒肺炎的价值分析]. *Journal of Emergency in Traditional Chinese Medicine[中国中医急症].*<br>11. (2019). Clinical study on shufeng jiedu capsule combined with cefoperazone sodium and sulbactam sodium in the treatment of community acquired pneumonia, 26(5), 413-415. doi: 10.19526/j.cnki.1005-8915.20190509<br>12. Liu, R. LI, X. L., Wang, L. Y., ... Li, J. S. (2018). Meta-analysis and GRADE Assessment of Shufeng Jiedu Capsule（疏风解毒胶囊）Combined with Antibiotics in Treating Community Acquired Pneumonia. *Journal of Traditional Chinese Medicine*, 59(19), 1656-1660<br>13. Liu, R. LI, X. L., Wang, L. Y., ... Li, J. S. (2018). Meta-analysis and GRADE Assessment of Shufeng Jiedu Capsule（疏风解毒胶囊）Combined with Antibiotics in Treating Community Acquired Pneumonia[疏风解毒胶囊联合抗生素治疗社区获得性肺炎的 Meta 分析与 GRADE 评价]. *Journal of Traditional Chinese Medicine [中医杂志]*, 59(19), 1656-1660.<br>14. Pan, D. M. (2017). Clinical efficacy of Shufeng Jiedu capsule in the treatment of community-acquired pneumonia and its influence on the duration of antibiotic use[疏风解毒胶囊治疗社区获得性肺炎临床疗效及对抗生素使用时间的影响探究]. *Journal of Medical Information(Xi-an) [医学信息(西安)]*, 30(16), 118-119<br>15. Liu, W. (2016). Clinical effect of Shufengjiedu capsuleS in the treatment of 50 cases of community-acquired pneumonia[疏风解毒胶囊治疗社区获得性肺炎 50 例临床疗效]. *Shanghai Medical & Pharmaceutical Journal [上海医药]*, 37(08), 27-28<br>16. Li, Y., Jia, M. Y., Zhang, J., ... Wang, X. J. (2015). Evaluation on clinical efficacy of Shufeng Jiedu Capsule on community-acquired pneumonia and its influence on therapeutic time of antibiotic[疏风解毒胶囊治疗社区获得性肺炎临床疗效及对抗生素使用时间的影响]. *China Journal of Traditional Chinese Medicine and Pharmacy[中华中医药杂志]*, 30(6), 2239-2242
Different participants (n=7)	1. Cao, L. F., Tang, C., Xia, j., Qu, X. K. (2019). Observation of the effect of Shufeng Jiedu Capsules for AECOPD complicated with pulmonary infection[疏风解毒胶囊治疗 AECOPD 合并肺部感染的疗效观察]. *Journal of Emergency in Traditional Chinese Medicine [中国中医急症]*, 28(9), 1636-1638<br>2. Liu, Q. (2018). Clinical effect of Shufeng Jiedu capsule combined with roxithromycin for pneumonia in elderly patients [疏风解毒胶囊联合

	罗红霉素治疗老年肺炎患者的效果观察]. *China Health Care & Nutrition[中国保健营养]*, 28(12), 206. doi: 10.3969/j.issn.1004-7484.2018.12.191<br>3. Li, J. F., Chen, C. S., Jiang, K. (2018). Clinical effectiveness of Shufeng Jiedu Capsules for 129 patients with post-cold cough and its expression of related inflammatory factors [疏风解毒胶囊治疗 129 例感冒后咳嗽患者的临床疗效分析及其对相关炎症因子的表达研究]. *Journal of Clinical Pulmonary Medicine [临床肺科杂志]*, 23(07), 1346-1348<br>4. Liu, Q. (2018). The effectiveness of Shufeng Jiedu Capsules combined with roxithromycin for pneumonia in elderly patients [疏风解毒胶囊联合罗红霉素治疗老年肺炎患者的效果观察]. *China Health Care & Nutrition [中国保健营养]*, 28(12), 206. doi: 10.3969/j.issn.1004-7484.2018.12.191<br>5. Du, Y. P., Ma, Y. M., Chen, Z. X. (2016). Clinical effectiveness of Shufeng Jiedu Capsules combined with azithromycin for pneumonia in elderly patients [疏风解毒胶囊联合阿奇霉素治疗老年肺炎患者的效果观察]. *China Health Care & Nutrition [中国保健营养]*, 26(15), 241-242<br>6. Wang, C. L., Wu, X. J., Xue, M. M., ... Tao, Z. G. (2014). Combination of "Shufeng Jiedu Capsule" and antibiotics for the treatment of diabetes combined with pulmonary infection. [疏风解毒胶囊联合抗生素治疗糖尿病合并肺部感染临床观察]. *Shanghai Journal of Traditional Chinese Medicine [上海中医药杂志]*, 48(11), 39-41, 48<br>7. Wang, C. L., Wu, X. J., Xue, M. M., ... Tao, Z. G. (2014). Combination of Shufeng Jiedu Capsules and antibiotics for the treatment of diabetes combined with pulmonary infection. [疏风解毒胶囊联合抗生素治疗糖尿病合并肺部感染临床观察]. *Shanghai Journal of Traditional Chinese Medicine [上海中医药杂志]*, 48(11), 39-41
Not use SFJD (n=1)	1. Liu, X. J. (2008). Clinical effectiveness of self-made Shufeng Qingre Jiedu decoction for children with bronchial pneumonia [自拟疏风清热解毒汤治疗小儿支气管肺炎效果观察]. *Chinese Journal of Rural Medicine and Pharmacy [中国乡村医药]*, 15(7)
Duplication (n=11)	1. Chen, J., Lin, S., Niu, C., & Xiao, Q. (2021). Clinical evaluation of Shufeng Jiedu Capsules combined with umifenovir (Arbidol) in the treatment of common-type COVID-19: a retrospective study. *EXPERT REVIEW OF RESPIRATORY MEDICINE*, 15(2), 257-265. doi: 10.1080/17476348.2020.1822741<br>2. CHEN, L., LIU, F., WU, J., SONG, H., XIA, J., SHENG, B.,... CHEN, Y. (2020). Clinical efficacy of Shufeng Jiedu Capsule combined with western medicine in treatment of common COVID-19 patients by retrospective analysis. *Chinese Journal of Experimental Traditional Medical Formulae*, 14-20

3. Qu, X. K., Hao, S. L., Ma, J. H., Wei, G. Y., Song, K. Y., Tang, C.,... Du, W. J. (2020). Observation on clinical effect of Shufeng Jiedu Capsule combined with Arbidol Hydrochloride Capsule in treatment of COVID-19. *Chinese Traditional and Herbal Drugs*, 51(5), 1167-1170. doi: 10.7501/j.issn.0253-2670.2020.05.011
4. Botai, W. (2020). Efficacy of Shufeng Jiedu Capsule combined with moxifloxacin in the treatment of community-acquired pneumonia. *Journal of Clinical Medicine in Practice[实用临床医药杂志]*, 24(19), 41-44
5. Wu, X., Mao, Y., & Wu, H. M. (2019). Clinical study on shufeng jiedu capsule combined with cefoperazone sodium and sulbactam sodium in the treatment of community acquired pneumonia. *Chinese Journal of Pharmaceutical Biotechnology*, 26(5), 413-415. doi: 10.19526/j.cnki.1005-8915.20190509
6. Wu, X., Mao, Y., & Wu, H. M. (2019). Clinical study on shufeng jiedu capsule combined with cefoperazone sodium and sulbactam sodium in the treatment of community acquired pneumonia. *Chinese Journal of Pharmaceutical Biotechnology*, 26(5), 413-415. doi: 10.19526/j.cnki.1005-8915.20190509
7. Wu, X., Mao, Y., & Wu, H. M. (2019). Clinical study on shufeng jiedu capsule combined with cefoperazone sodium and sulbactam sodium in the treatment of community acquired pneumonia. *Chinese Journal of Pharmaceutical Biotechnology*, 26(5), 413-415. doi: 10.19526/j.cnki.1005-8915.20190509
8. X Wu, Y. M. H. W. (2019). Clinical study on shufeng jiedu capsule combined with cefoperazone sodium and sulbactam sodium in the treatment of community acquired pneumonia[疏风解毒胶囊联合头孢哌酮钠舒巴坦钠治疗社区获得性肺炎的临床研究]. *Chinese journal of pharmaceutical biotechnology.*
9. Wu, X., Mao, Y., & Wu, H. M. (2019). Clinical study on shufeng jiedu capsule combined with cefoperazone sodium and sulbactam sodium in the treatment of community acquired pneumonia. *Chinese Journal of Pharmaceutical Biotechnology*, 26(5), 413-415. doi: 10.19526/j.cnki.1005-8915.20190509
10. Zhang, S., Li, T., Mao, X. D., ... Fan, Y. H. (2016). Clinical observation of Shufeng Jiedu Capsules combined with levofloxacin for community-acquired pneumonia without increased peripheral blood leukocytes[疏风解毒胶囊联合左氧氟沙星治疗不伴有外周血白细胞升高的社区获得性肺炎的临床疗效观察]. Paper presented at the *The 14th National Symposium on the Prevention and Treatment of Respiratory System Diseases with Integrated Traditional Chinese and Western Medicine[第十四次全国中西医结合防治呼吸系统疾病学术研讨会]*, Xingyi, Guizhou.
11. Wang, C. L., Wu, X. J., Xue, M. M., ... Tao, Z. G. (2014). Combination of Shufeng Jiedu Capsules and antibiotics for the treatment of

diabetes combined with pulmonary infection. [疏风解毒胶囊联合抗生素治疗糖尿病合并肺部感染临床观察]. *Shanghai Journal of Traditional Chinese Medicine [上海中医药杂志]*, 48(11), 39-41, 48

Supplementary Appendix A3: Summary of composition characteristics of preparations in all included articles

Study ID	Formulation	Source	Species, concentration	Quality control reported? (Y/N)	Chemical analysis reported? (Y/N)
Tang et al. (2021)	Shufeng Jiedu Capsules	Anhui Jiren Pharmaceutical Co., Ltd.	*Bupleurum chinense DC. [Apiaceae], Forsythia suspensa (Thunb.) Vahl [Oleaceae], Glycyrrhiza uralensis Fisch. ex DC. [Fabaceae], Isatis tinctoria subsp. tinctoria [Brassicaceae], Patrinia scabiosifolia f. scabiosifolia [Caprifoliaceae], Phragmites australis subsp. australis [Poaceae], Reynoutria japonica Houtt. [Polygonaceae], Verbena officinalis L. [Verbenaceae]*; 0.52g/capsule	Y - prepared according to Pharmacopedia of the People's Republic of China (2015) and National Medical Products Administration Standards YBZ05182019	N
Wang (2020)	Shufeng Jiedu Capsules	Anhui Jiren Pharmaceutical Co., Ltd.	*Bupleurum chinense DC. [Apiaceae], Forsythia suspensa (Thunb.) Vahl [Oleaceae], Glycyrrhiza uralensis Fisch. ex DC. [Fabaceae], Isatis tinctoria subsp. tinctoria [Brassicaceae], Patrinia scabiosifolia f. scabiosifolia [Caprifoliaceae], Phragmites australis subsp. australis [Poaceae], Reynoutria japonica Houtt. [Polygonaceae], Verbena officinalis L. [Verbenaceae]*; 0.52g/capsule	Y - prepared according to Pharmacopedia of the People's Republic of China (2015) and National Medical Products Administration Standards YBZ05182019	Y - *Bupleurum chinense DC. [Apiaceae]* contains Bupleurum saponins, phytosterols and other substances, which have the functions of relieving Qi and depression, dispersing fire and raising Yang;<br>The effective component of *Forsythia suspensa (Thunb.) Vahl [Oleaceae]* is forsythiol, which

					can inhibit the growth and reproduction of Staphylococcus aureus and typhoid bacillus.
Li DW et al. (2020a)	Shufeng Jiedu Capsules	N	*Bupleurum chinense DC. [Apiaceae], Forsythia suspensa (Thunb.) Vahl [Oleaceae], Glycyrrhiza uralensis Fisch. ex DC. [Fabaceae], Isatis tinctoria subsp. tinctoria [Brassicaceae], Patrinia scabiosifolia f. scabiosifolia [Caprifoliaceae], Phragmites australis subsp. australis [Poaceae], Reynoutria japonica Houtt. [Polygonaceae], Verbena officinalis L. [Verbenaceae]*	N	N
Li XY et al. (2020b)	Shufeng Jiedu Capsules	Anhui Jiren Pharmaceutical Co., Ltd.	*Bupleurum chinense DC. [Apiaceae], Forsythia suspensa (Thunb.) Vahl [Oleaceae], Glycyrrhiza uralensis Fisch. ex DC. [Fabaceae], Isatis tinctoria subsp. tinctoria [Brassicaceae], Patrinia scabiosifolia f. scabiosifolia [Caprifoliaceae], Phragmites australis subsp. australis [Poaceae], Reynoutria japonica Houtt. [Polygonaceae], Verbena officinalis L. [Verbenaceae]*; 0.52g/capsule	Y - prepared according to Pharmacopedia of the People's Republic of China (2015) and National Medical Products Administration Standards YBZ05182019	Y - the phenylethanol glycosides and iridoid glycosides contained in Shufeng Jiedu Capsules can inhibit the release of inflammatory factors
Guo et al. (2020)	Shufeng Jiedu Capsules	Anhui Jiren Pharmaceutical Co., Ltd.	*Bupleurum chinense DC. [Apiaceae], Forsythia suspensa (Thunb.) Vahl [Oleaceae], Glycyrrhiza uralensis Fisch. ex DC. [Fabaceae], Isatis tinctoria subsp. tinctoria [Brassicaceae], Patrinia scabiosifolia f. scabiosifolia [Caprifoliaceae], Phragmites australis subsp. australis [Poaceae], Reynoutria japonica Houtt. [Polygonaceae], Verbena officinalis L. [Verbenaceae]*; 0.52g/capsule	Y - prepared according to Pharmacopedia of the People's Republic of China (2015) and National Medical Products Administration Standards YBZ05182019	N
Pan et al. (2020)	Shufeng Jiedu Capsules	Anhui Jiren Pharmaceutical Co., Ltd.	*Bupleurum chinense DC. [Apiaceae], Forsythia suspensa (Thunb.) Vahl [Oleaceae], Glycyrrhiza uralensis Fisch. ex DC. [Fabaceae], Isatis tinctoria subsp. tinctoria [Brassicaceae], Patrinia*	Y - prepared according to Pharmacopedia of the People's Republic of China (2015) and	N

			scabiosifolia f. scabiosifolia [Caprifoliaceae], Phragmites australis subsp. australis [Poaceae], Reynoutria japonica Houtt. [Polygonaceae], Verbena officinalis L. [Verbenaceae]; 0.52g/capsule	National Medical Products Administration Standards YBZ05182019	
Wu et al. (2019)	Shufeng Jiedu Capsules	Anhui Jiren Pharmaceutical Co., Ltd.	*Bupleurum chinense DC. [Apiaceae], Forsythia suspensa (Thunb.) Vahl [Oleaceae], Glycyrrhiza uralensis Fisch. ex DC. [Fabaceae], Isatis tinctoria subsp. tinctoria [Brassicaceae], Patrinia scabiosifolia f. scabiosifolia [Caprifoliaceae], Phragmites australis subsp. australis [Poaceae], Reynoutria japonica Houtt. [Polygonaceae], Verbena officinalis L. [Verbenaceae]*; 0.52g/capsule	Y - prepared according to Pharmacopedia of the People's Republic of China (2015) and National Medical Products Administration Standards	N
Zhou et al. (2019)	Shufeng Jiedu Capsules	Anhui Jiren Pharmaceutical Co., Ltd.	*Bupleurum chinense DC. [Apiaceae], Forsythia suspensa (Thunb.) Vahl [Oleaceae], Glycyrrhiza uralensis Fisch. ex DC. [Fabaceae], Isatis tinctoria subsp. tinctoria [Brassicaceae], Patrinia scabiosifolia f. scabiosifolia [Caprifoliaceae], Phragmites australis subsp. australis [Poaceae], Reynoutria japonica Houtt. [Polygonaceae], Verbena officinalis L. [Verbenaceae]*; 0.52g/capsule	Y - prepared according to Pharmacopedia of the People's Republic of China (2015) and National Medical Products Administration Standards	N
Qu et al. (2019)	Shufeng Jiedu Capsules	Anhui Jiren Pharmaceutical Co., Ltd.	*Bupleurum chinense DC. [Apiaceae], Forsythia suspensa (Thunb.) Vahl [Oleaceae], Glycyrrhiza uralensis Fisch. ex DC. [Fabaceae], Isatis tinctoria subsp. tinctoria [Brassicaceae], Patrinia scabiosifolia f. scabiosifolia [Caprifoliaceae], Phragmites australis subsp. australis [Poaceae], Reynoutria japonica Houtt. [Polygonaceae], Verbena officinalis L. [Verbenaceae]*; 0.52g/capsule	Y - prepared according to Pharmacopedia of the People's Republic of China (2015) and National Medical Products Administration Standards	N
Wei et al.	Shufeng	Anhui Jiren	*Bupleurum chinense DC. [Apiaceae], Forsythia suspensa (Thunb.)*	N	N

(2016)	Jiedu Capsules	Pharmaceutical Co., Ltd.	*Vahl [Oleaceae], Glycyrrhiza uralensis Fisch. ex DC. [Fabaceae], Isatis tinctoria subsp. tinctoria [Brassicaceae], Patrinia scabiosifolia f. scabiosifolia [Caprifoliaceae], Phragmites australis subsp. australis [Poaceae], Reynoutria japonica Houtt. [Polygonaceae], Verbena officinalis L. [Verbenaceae]*		
Yao et al. (2016)	Shufeng Jiedu Capsules	Anhui Jiren Pharmaceutical Co., Ltd.	*Bupleurum chinense DC. [Apiaceae], Forsythia suspensa (Thunb.) Vahl [Oleaceae], Glycyrrhiza uralensis Fisch. ex DC. [Fabaceae], Isatis tinctoria subsp. tinctoria [Brassicaceae], Patrinia scabiosifolia f. scabiosifolia [Caprifoliaceae], Phragmites australis subsp. australis [Poaceae], Reynoutria japonica Houtt. [Polygonaceae], Verbena officinalis L. [Verbenaceae]*; 0.52g/capsule	N	N
Zhang et al. (2016)	Shufeng Jiedu Capsules	N	*Bupleurum chinense DC. [Apiaceae], Forsythia suspensa (Thunb.) Vahl [Oleaceae], Glycyrrhiza uralensis Fisch. ex DC. [Fabaceae], Isatis tinctoria subsp. tinctoria [Brassicaceae], Patrinia scabiosifolia f. scabiosifolia [Caprifoliaceae], Phragmites australis subsp. australis [Poaceae], Reynoutria japonica Houtt. [Polygonaceae], Verbena officinalis L. [Verbenaceae]*	N	N
Zhu et al. (2016)	Shufeng Jiedu Capsules	Anhui Jiren Pharmaceutical Co., Ltd.	*Bupleurum chinense DC. [Apiaceae], Forsythia suspensa (Thunb.) Vahl [Oleaceae], Glycyrrhiza uralensis Fisch. ex DC. [Fabaceae], Isatis tinctoria subsp. tinctoria [Brassicaceae], Patrinia scabiosifolia f. scabiosifolia [Caprifoliaceae], Phragmites australis subsp. australis [Poaceae], Reynoutria japonica Houtt. [Polygonaceae], Verbena officinalis L. [Verbenaceae]*; 0.52g/capsule	Y - prepared according to National Medical Products Administration Standards	N
Wang	Shufeng	N	*Bupleurum chinense DC. [Apiaceae], Forsythia suspensa (Thunb.)*	N	N

(2016)	Jiedu Capsules		*Vahl [Oleaceae], Glycyrrhiza uralensis Fisch. ex DC. [Fabaceae], Isatis tinctoria subsp. tinctoria [Brassicaceae], Patrinia scabiosifolia f. scabiosifolia [Caprifoliaceae], Phragmites australis subsp. australis [Poaceae], Reynoutria japonica Houtt. [Polygonaceae], Verbena officinalis L. [Verbenaceae]*		
Li et al. (2015)	Shufeng Jiedu Capsules	Anhui Jiren Pharmaceutical Co., Ltd.	*Bupleurum chinense DC. [Apiaceae], Forsythia suspensa (Thunb.) Vahl [Oleaceae], Glycyrrhiza uralensis Fisch. ex DC. [Fabaceae], Isatis tinctoria subsp. tinctoria [Brassicaceae], Patrinia scabiosifolia f. scabiosifolia [Caprifoliaceae], Phragmites australis subsp. australis [Poaceae], Reynoutria japonica Houtt. [Polygonaceae], Verbena officinalis L. [Verbenaceae]*	N	N
Zou (2015)	Shufeng Jiedu Capsules	Anhui Jiren Pharmaceutical Co., Ltd.	*Bupleurum chinense DC. [Apiaceae], Forsythia suspensa (Thunb.) Vahl [Oleaceae], Glycyrrhiza uralensis Fisch. ex DC. [Fabaceae], Isatis tinctoria subsp. tinctoria [Brassicaceae], Patrinia scabiosifolia f. scabiosifolia [Caprifoliaceae], Phragmites australis subsp. australis [Poaceae], Reynoutria japonica Houtt. [Polygonaceae], Verbena officinalis L. [Verbenaceae]*	N	N
Zhang et al. (2014)	Shufeng Jiedu Capsules	Anhui Jiren Pharmaceutical Co., Ltd.	*Bupleurum chinense DC. [Apiaceae], Forsythia suspensa (Thunb.) Vahl [Oleaceae], Glycyrrhiza uralensis Fisch. ex DC. [Fabaceae], Isatis tinctoria subsp. tinctoria [Brassicaceae], Patrinia scabiosifolia f. scabiosifolia [Caprifoliaceae], Phragmites australis subsp. australis [Poaceae], Reynoutria japonica Houtt. [Polygonaceae], Verbena officinalis L. [Verbenaceae]*	N	N

Notes: Concentration of each species in the preparations belong to the core technology of the pharmaceutical companies, so they did not report the grams of each composition.

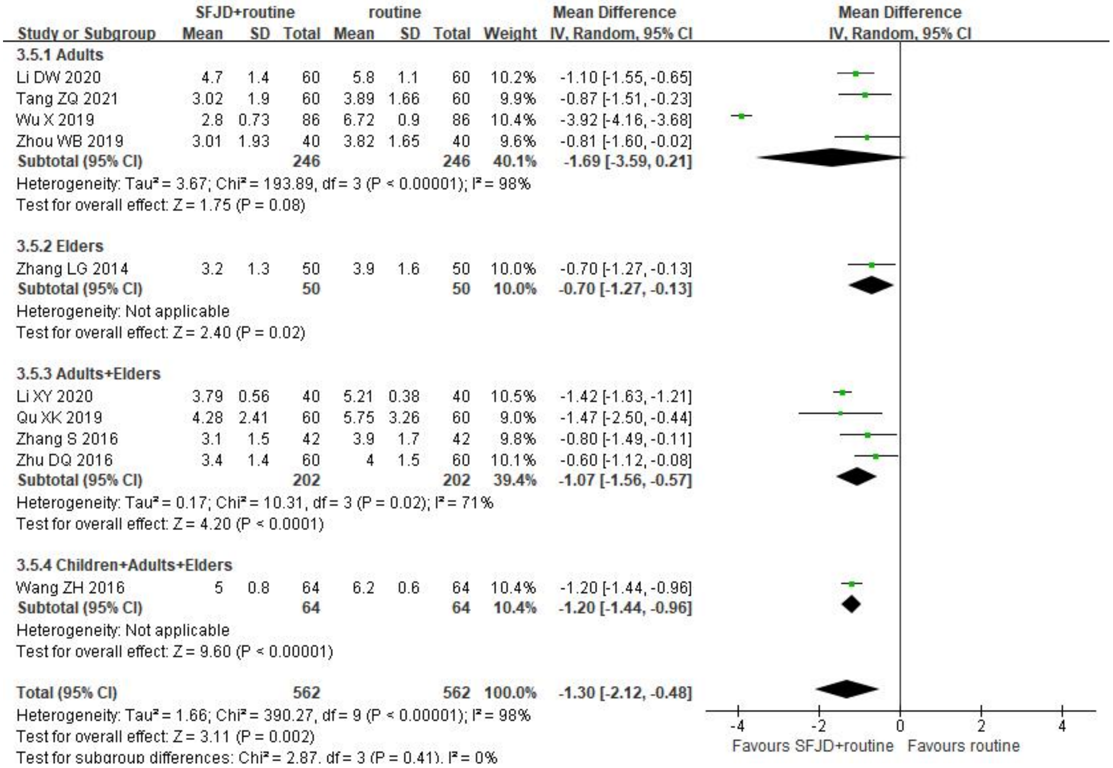

Supplementary Appendix A4: Forest plot of resolution time of phlegm (days) stratified by age. Comparison: SFJD plus routine treatment vs. routine treatment. SFJD: Shufeng Jiedu.

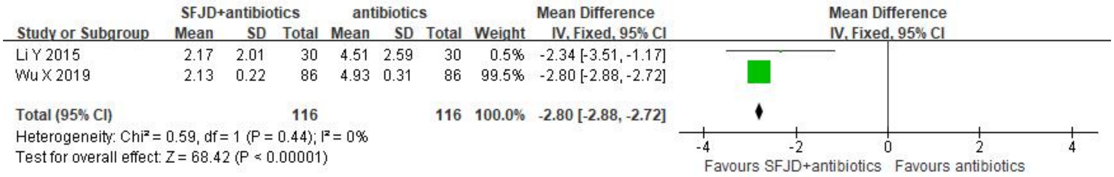

Supplementary Appendix A5: Forest plot of resolution time of shortness of breath (days). Comparison: SFJD plus antibiotics vs. Antibiotics. SFJD: Shufeng Jiedu.

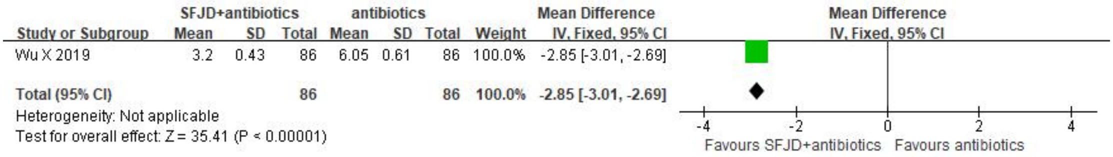

Supplementary Appendix A6: Forest plot of resolution time of chest pain (days). Comparison: SFJD plus antibiotics vs. Antibiotics. SFJD: Shufeng Jiedu.

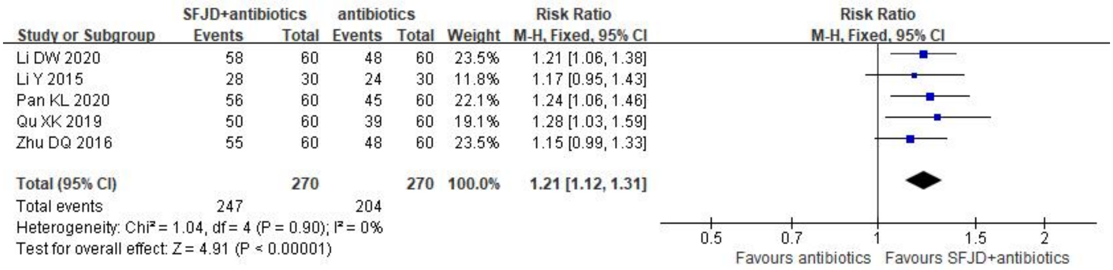

Supplementary Appendix A7 Forest plot of improvement rate of chest radiograph. Comparison: SFJD plus routine treatment vs. routine treatment. SFJD: Shufeng Jiedu.

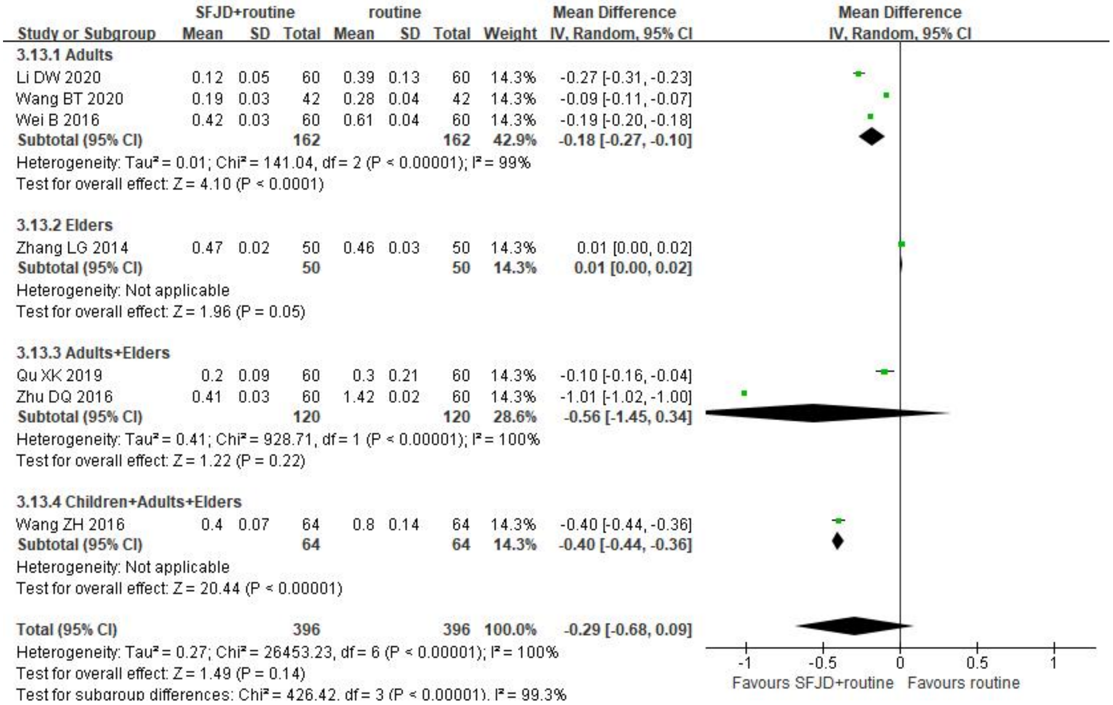

Supplementary Appendix A8: Forest plot of procalcitonin (PCT) stratified by age (ng/mL). Comparison: SFJD plus routine treatment vs. routine treatment. SFJD: Shufeng Jiedu. SFJD: Shufeng Jiedu.

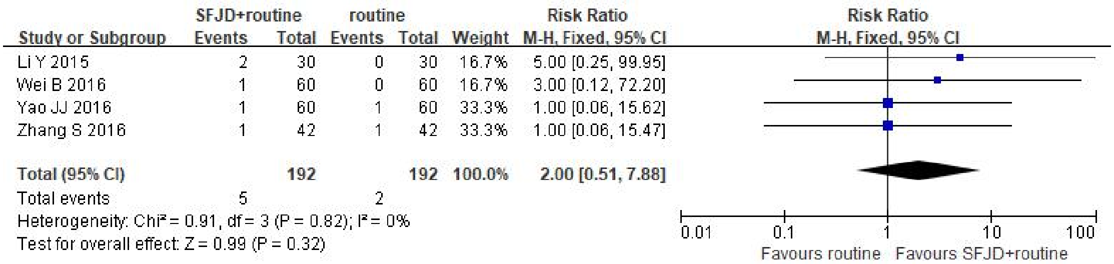

Supplementary Appendix A9: Forest plot of adverse event-nausea. Comparison: SFJD plus routine treatment vs. routine treatment. SFJD: Shufeng Jiedu. SFJD: Shufeng Jiedu.

Study or Subgroup	SFJD+antibiotics Mean	SD	Total	antibiotics Mean	SD	Total	Weight	Mean Difference IV, Random, 95% CI
Guo GH 2020	3.26	1.08	46	3.85	1.21	46	11.5%	-0.59 [-1.06, -0.12]
Li Y 2015	2.31	3.11	30	3.53	3.92	30	7.7%	-1.22 [-3.01, 0.57]
Qu XK 2019	2.34	1.94	60	3.07	1.81	60	11.1%	-0.73 [-1.40, -0.06]
Tang ZQ 2021	2.79	0.79	60	3.53	1.21	60	11.7%	-0.74 [-1.11, -0.37]
Wang ZH 2016	2.4	0.9	64	4.2	1.5	64	11.6%	-1.80 [-2.23, -1.37]
Wei B 2016	2.08	1.79	60	2.67	1.89	60	11.1%	-0.59 [-1.25, 0.07]
Wu X 2019	1.59	0.32	86	4.75	0.81	86	11.9%	-3.16 [-3.34, -2.98]
Yao JJ 2016	2.6	0.6	57	4.8	1.1	58	11.8%	-2.20 [-2.52, -1.88]
Zhou WB 2019	2.81	0.9	40	3.52	1.22	40	11.5%	-0.71 [-1.18, -0.24]
**Total (95% CI)**			**503**			**504**	**100.0%**	**-1.32 [-2.15, -0.49]**

Heterogeneity: Tau² = 1.51; Chi² = 293.39, df = 8 (P < 0.00001); I² = 97%
Test for overall effect: Z = 3.11 (P = 0.002)

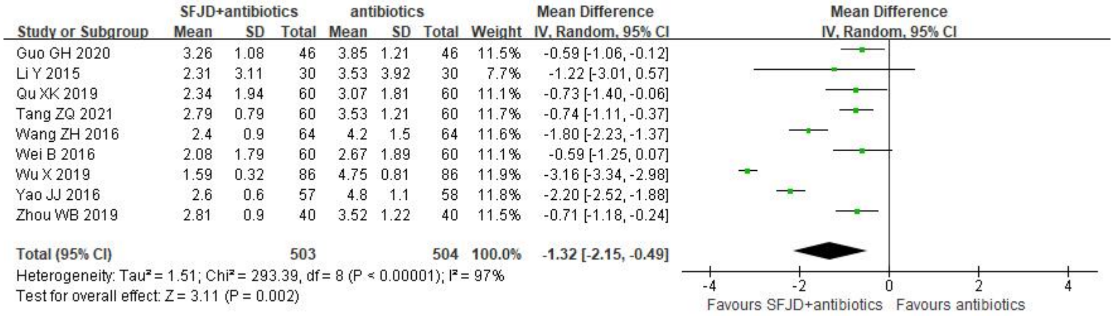

Supplementary Appendix A10a: Forest plot of resolution time of fever (days) (Omission of high risk of bias). Comparison: SFJD plus routine treatment vs. routine treatment. SFJD: Shufeng Jiedu capsule.

Study or Subgroup	SFJD+antibiotics Mean	SD	Total	antibiotics Mean	SD	Total	Weight	Mean Difference IV, Fixed, 95% CI
Guo GH 2020	5.64	1.32	46	6.89	1.73	46	13.1%	-1.25 [-1.88, -0.62]
Li Y 2015	4.03	5.67	30	6.49	5.03	30	0.7%	-2.46 [-5.17, 0.25]
Qu XK 2019	5.92	2.54	60	6.18	2.26	60	7.0%	-0.26 [-1.12, 0.60]
Tang ZQ 2021	4.83	2.15	60	5.78	1.68	60	10.9%	-0.95 [-1.64, -0.26]
Wang ZH 2016	5.6	1.4	64	6.4	1.6	64	19.1%	-0.80 [-1.32, -0.28]
Wei B 2016	5.71	1.91	60	6.49	1.73	60	12.2%	-0.78 [-1.43, -0.13]
Yao JJ 2016	4.8	1.1	57	5.7	1.2	58	29.3%	-0.90 [-1.32, -0.48]
Zhou WB 2019	4.91	2.12	40	5.81	1.6	40	7.7%	-0.90 [-1.72, -0.08]
**Total (95% CI)**			**417**			**418**	**100.0%**	**-0.88 [-1.11, -0.66]**

Heterogeneity: Chi² = 4.86, df = 7 (P = 0.68); I² = 0%
Test for overall effect: Z = 7.61 (P < 0.00001)

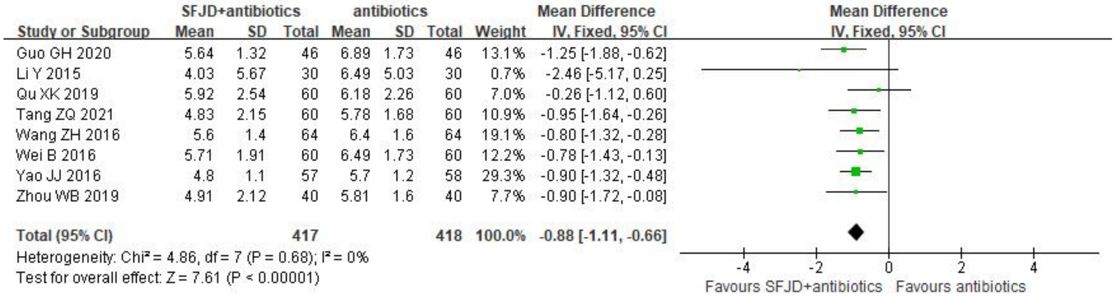

Supplementary Appendix A10b: Forest plot of resolution time of cough (days) (Omission of high risk of bias). Comparison: SFJD plus routine treatment vs. routine treatment. SFJD: Shufeng Jiedu capsule.

Study or Subgroup	SFJD+antibiotics Mean	SD	Total	antibiotics Mean	SD	Total	Weight	Mean Difference IV, Random, 95% CI
Qu XK 2019	4.28	2.41	60	5.75	3.26	60	19.0%	-1.47 [-2.50, -0.44]
Tang ZQ 2021	3.02	1.9	60	3.89	1.66	60	20.0%	-0.87 [-1.51, -0.23]
Wang ZH 2016	5	0.8	64	6.2	0.6	64	20.6%	-1.20 [-1.44, -0.96]
Wu X 2019	2.8	0.73	86	6.72	0.9	86	20.6%	-3.92 [-4.16, -3.68]
Zhou WB 2019	3.01	1.93	40	3.82	1.65	40	19.7%	-0.81 [-1.60, -0.02]
**Total (95% CI)**			**310**			**310**	**100.0%**	**-1.67 [-3.23, -0.11]**

Heterogeneity: Tau² = 3.05; Chi² = 280.68, df = 4 (P < 0.00001); I² = 99%
Test for overall effect: Z = 2.10 (P = 0.04)

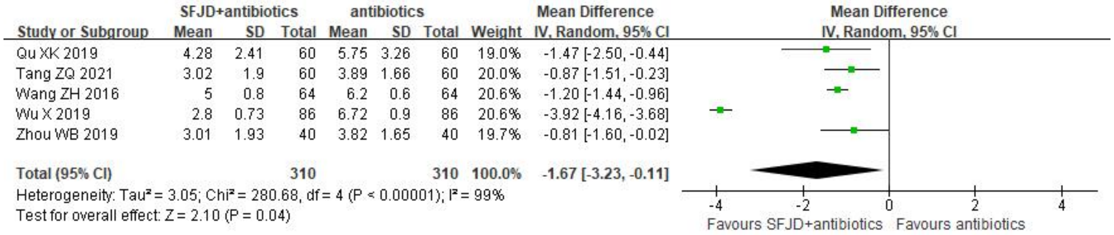

Supplementary Appendix A10c: Forest plot of resolution time of phlegm (days) (Omission of high risk of bias). Comparison: SFJD plus routine treatment vs. routine treatment. SFJD: Shufeng Jiedu capsule.

Study or Subgroup	SFJD+antibiotics Mean	SD	Total	antibiotics Mean	SD	Total	Weight	Mean Difference IV, Random, 95% CI
Guo GH 2020	5.83	1.48	46	7.03	2.17	46	15.4%	-1.20 [-1.96, -0.44]
Li Y 2015	4.08	5.19	30	5.99	8.37	30	6.8%	-1.91 [-5.43, 1.61]
Qu XK 2019	3.83	2.47	60	5.28	3.58	60	14.4%	-1.45 [-2.55, -0.35]
Tang ZQ 2021	3.68	1.75	60	4.25	1.08	60	15.9%	-0.57 [-1.09, -0.05]
Wang ZH 2016	4.7	1.5	64	6.8	2.2	64	15.6%	-2.10 [-2.75, -1.45]
Wu X 2019	2.74	0.26	86	5.98	0.53	86	16.4%	-3.24 [-3.36, -3.12]
Zhou WB 2019	3.71	1.81	40	4.4	1.12	40	15.6%	-0.69 [-1.35, -0.03]
**Total (95% CI)**			**386**			**386**	**100.0%**	**-1.58 [-2.77, -0.38]**

Heterogeneity: Tau² = 2.27; Chi² = 180.48, df = 6 (P < 0.00001); I² = 97%
Test for overall effect: Z = 2.59 (P = 0.010)

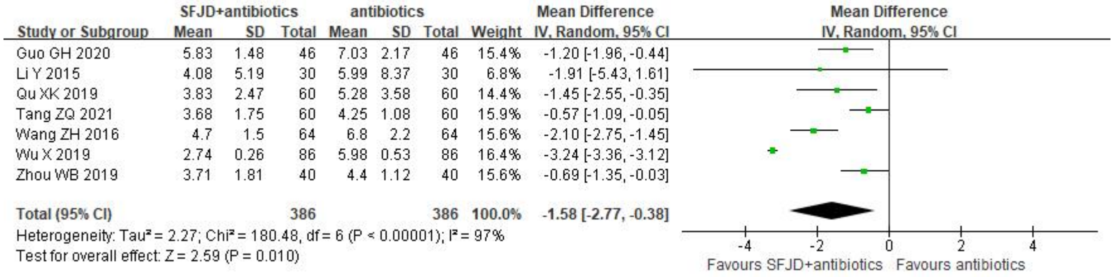

Supplementary Appendix A10d: Forest plot of resolution time of pulmonary crepitations (days) (Omission of high risk of bias). Comparison: SFJD plus routine treatment vs. routine treatment. SFJD: Shufeng Jiedu capsule.

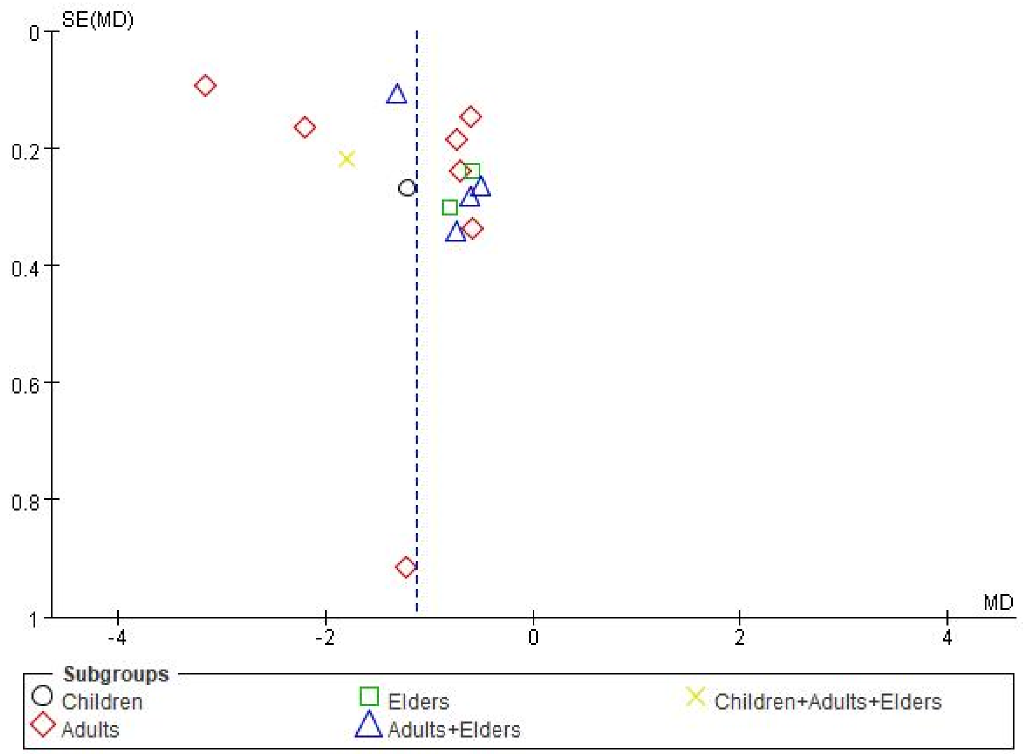

Supplementary Appendix A11a: Funnel plot of comparison: SFJD plus routine treatment vs. routine treatment, outcome: duration of fever. SFJD: Shufeng Jiedu.

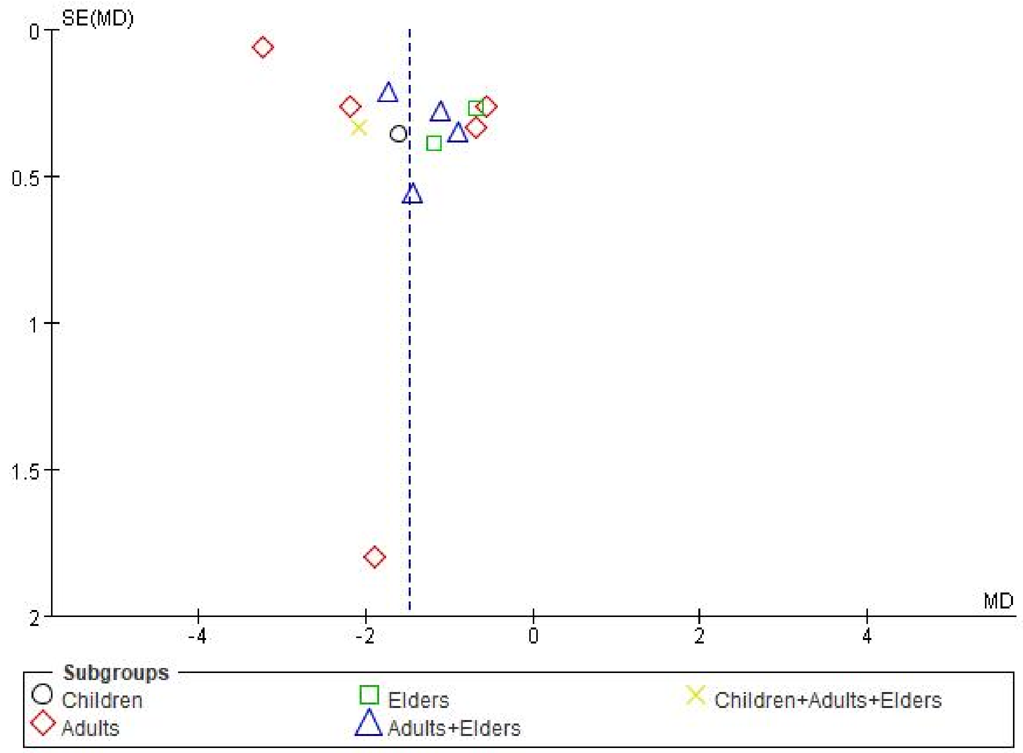

Supplementary Appendix A11b: Funnel plot of comparison: SFJD plus routine treatment vs. routine treatment, outcome: duration of pulmonary crepitations. SFJD: Shufeng Jiedu.

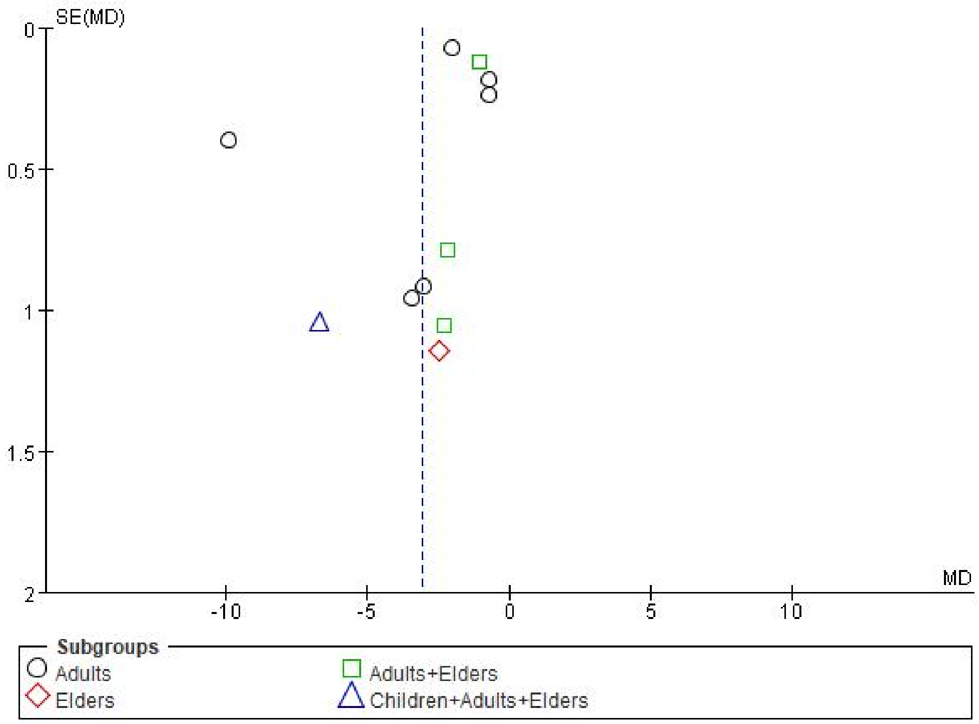

Supplementary Appendix A11c: Funnel plot of comparison: SFJD plus routine treatment vs. routine treatment, outcome: C-reactive protein (CRP). SFJD: Shufeng Jiedu.
